# DeepTaxa: a hybrid CNN-BERT framework for 16S rRNA taxonomic classification

**DOI:** 10.1093/bioadv/vbag166

**Published:** 2026-06-12

**Authors:** Rana Salah, Khlood R AbdElaal, Lobna Ghonaim, Olaitan I Awe, Ahmed Moustafa

**Affiliations:** Systems Genomics Lab, The American University, Cairo, New Cairo, Egypt; Biotechnology Graduate Program, The American University, Cairo, New Cairo, Egypt; Systems Genomics Lab, The American University, Cairo, New Cairo, Egypt; Biotechnology Graduate Program, The American University, Cairo, New Cairo, Egypt; Egypt Center for Research and Regenerative Medicine (ECRRM), Cairo, Egypt; Systems Genomics Lab, The American University, Cairo, New Cairo, Egypt; Biotechnology Graduate Program, The American University, Cairo, New Cairo, Egypt; African Society for Bioinformatics and Computational Biology, Cape Town, South Africa; Systems Genomics Lab, The American University, Cairo, New Cairo, Egypt; Biotechnology Graduate Program, The American University, Cairo, New Cairo, Egypt; Egypt Center for Research and Regenerative Medicine (ECRRM), Cairo, Egypt; Department of Biology, The American University, Cairo, New Cairo, Egypt

## Abstract

**Motivation:**

Accurate species-level classification of prokaryotic 16S rRNA sequences remains difficult: existing tools rely on exact alignment, k-mer heuristics, or phylogenetic placement and are limited by incomplete reference databases. Deep learning approaches in microbial genomics have focused largely on whole-genome metagenomics, leaving 16S taxonomy under-supported.

**Results:**

We present DeepTaxa, a hybrid CNN-BERT framework that pairs a multiscale CNN with a transformer trained from scratch on the DNABERT-2 BPE vocabulary, producing parallel rank-specific predictions across the seven Linnean ranks. On the Greengenes2 2024.09 test set, DeepTaxa achieves species-level accuracy of 92.96% and F1 of 0.9212 (3-seed mean; cross-seed standard deviation ≤0.0008 F1 at every rank), with F1 above 0.99 from domain through class and a species-level expected calibration error of 0.0242. DeepTaxa exceeds DADA2 (90.05%) and QIIME 2 (85.01%) at the species rank on the same held-out test set, with larger gains over the k-mer-based classifiers SINTAX and Kraken 2. Performance degrades smoothly with decreasing training-set similarity (species F1 from 0.95 to 0.45), and a dedicated V3–V4 amplicon checkpoint reaches 87.55% species accuracy from an approximately 420 bp window.

**Availability and implementation:**

Source code, trained checkpoints for full-length 16S and V3–V4 amplicons, curated datasets, and reproducible workflows are publicly available at github.com/systems-genomics-lab/deeptaxa and huggingface.co/systems-genomics-lab/deeptaxa.

## Introduction

Taxonomic classification has advanced substantially over the past two decades, particularly with the advent of high-throughput sequencing technologies that allow the characterization of microbial communities at high throughput (Caporaso *et al.* 2011). It is central to biological research in microbiome studies, disease diagnostics, and environmental monitoring (Janda and Abbott 2007). A widely used method for studying microbial communities is 16S rRNA sequencing, which targets a conserved bacterial gene, with species-specific variations that enable bacterial and archaeal identification. Its affordability, scalability, and well-established analytical workflows have made it a popular choice for large-scale studies (Caporaso *et al.* 2011). Despite the rise of shotgun metagenomics, 16S rRNA surveys remain relevant because of their broad coverage, deeper sampling per sample, and substantially lower sequencing and computational costs (Clarridge 2004; Matchado *et al.* 2024). Curated databases such as Greengenes2 (McDonald *et al.* 2024), SILVA (Quast *et al.* 2013), and the Ribosomal Database Project (RDP) (Cole *et al.* 2009) offer greater species-level coverage than whole-genome references, making 16S metabarcoding a reliable and scalable approach, particularly in resource-limited settings or publicly funded projects (Matchado *et al.* 2024). These advantages have made 16S sequencing central to large-scale initiatives such as the Human Microbiome Project (Turnbaugh *et al.* 2007) and the Earth Microbiome Project (Gilbert *et al.* 2014). Continued development of tools that handle 16S data effectively is necessary to fully exploit this resource and to support the discovery of new species across diverse environments.

### Traditional computational approaches

Early computational methods for taxonomic classification relied heavily on sequence alignment techniques. BLAST (Altschul *et al.* 1990) and MEGAN (Huson *et al.* 2007) have long been foundational in taxonomy assignment, using sequence similarity to assign taxonomic labels. However, these tools often struggle to handle complex, large-scale datasets, to distinguish closely related species, and to process chimeric or short, error-prone sequences typical of 16S rRNA data (Quince *et al.* 2017). Other alignment-based approaches, such as MMseqs2 (Steinegger and Söding 2017), Minimap2 (Li 2018), and VSEARCH (Rognes *et al.* 2016), are frequently employed for their speed and sensitivity, although they do not directly perform taxonomic classification. Instead, they are used in preprocessing steps, where their outputs are fed into downstream classifiers like Kraken (Wood and Salzberg 2014) or Centrifuge (Kim *et al.* 2016). To address these limitations, k-mer-based approaches like CLARK (Ounit *et al.* 2015) and Kraken 2 (Wood *et al.* 2019) were developed, offering faster classification by analyzing the frequency of k-length subsequences. However, these methods often require extensive reference databases and may perform poorly for novel, rare, or uncultured species, leading to potential misidentifications and misrepresentation of microbial diversity, as these species are underrepresented in curated reference databases. These traditional pipelines are further hindered by high dimensionality, data sparsity, and the compositional nature of microbiome data (Roy *et al.* 2024).

In addition to standalone classifiers, several modular pipelines have been developed to support microbiome analysis. QIIME 2 (Bolyen *et al.* 2019), a widely adopted platform, integrates various tools, including alignment- and k-mer-based classifiers, for end-to-end microbiome data processing. It provides reproducibility, plugin flexibility, and interactive visualizations, making it a central hub for many researchers. Similarly, SINTAX (Edgar 2016) offers a non-Bayesian taxonomy classifier based on k-mer profiles. It is often used for rapid, reference-based assignments, particularly in large-scale 16S rRNA studies. However, such workflows still rely heavily on the performance of their underlying classifiers and are subject to limitations in reference database coverage and species-level resolution.

Taxonomic classification is limited by incomplete reference databases. Reference-based methods can only detect species already represented in genomic catalogs, leaving much of the microbial “dark matter” unclassified (Blanco-Míguez *et al.* 2023). The databases often lack coverage or quality, reducing sensitivity and specificity. Although large-scale assembly efforts have revealed thousands of novel genomes, many sequence reads remain unclassified or are assigned only to broad taxonomic levels (Breitwieser *et al.* 2019). Because supervised and reference-based classification methods depend on labeled reference sequences or taxonomies, these gaps directly hinder accurate identification, especially for novel or rare taxa. These limitations motivate the development of more flexible, data-driven models that learn directly from sequence patterns.

To overcome these shortcomings, deep learning (DL) models can learn high-dimensional sequence features directly from sequence representations and avoid explicit alignment or k-mer matching at inference time, although supervised models still depend on labeled reference taxonomies during training. These models have shown promise in a range of metagenomic tasks, including binning, functional annotation, and phenotype prediction, with potential to extend to taxonomic classification pipelines (Roy *et al.* 2024).

### Advancements in deep learning for taxonomic classification

The integration of DL into microbiome analysis has expanded the scope of taxonomic classification by enabling models to learn complex patterns directly from genetic data. Convolutional Neural Networks (CNNs), in particular, have gained prominence because they can automatically extract local sequence features without requiring handcrafted descriptors. For example, DeepMicrobes (Liang *et al.* 2020) uses a Bidirectional Long Short-Term Memory (BiLSTM) network with self-attention over k-mer embeddings for taxonomic classification, showing strong performance on noisy, real-world data; however, BiLSTM-only and CNN-only sequence models can be limited in their ability to capture long-range dependencies efficiently (Ji *et al.* 2021). This limitation motivated the development of transformer-based models (Vaswani *et al.* 2017), which model global dependencies through self-attention. Notable examples include BERTax (Mock *et al.* 2022), which uses Bidirectional Encoder Representations from Transformers (BERT) (Devlin *et al.* 2019) for taxonomic classification. Domain-adapted BERT variants such as DNABERT (Ji *et al.* 2021) extend the architecture to genomic sequences, and BarcodeBERT (Arias *et al.* 2026) applies deep contextual embeddings to insect DNA barcodes rather than 16S rRNA. These models illustrate the growing role of transformer-based architectures in nucleotide-sequence analysis.

Beyond the CNN and transformer architectures above, related sequence models have also been explored for nucleotide data, including GROVER (Sanabria *et al.* 2024), a transformer-based DNA language model trained on the human genome with a Byte Pair Encoding (BPE) tokenizer that demonstrates how subword tokenization can capture contextual information in nucleotide sequences.

Despite the growing interest in hybrid DL architectures, their application to microbiome taxonomic prediction remains limited, particularly for 16S rRNA data. Roy et al. (2024) identify DETIRE (Miao *et al.* 2023) as the sole hybrid model that combines graph-based embeddings with CNNs and BiLSTMs to classify viral sequences. However, DETIRE’s focus on viral metagenomes and its lack of transformer integration leave 16S classification without an architecture that captures both local features and long-range dependencies in the same pass.

A second concern is that, although the broader field of DL in metagenomics has seen rapid innovation, only a limited number of models have been explicitly developed for 16S rRNA-based taxonomic classification, as the review by Roy *et al*. (2024) notes. As our focus is limited to 16S applications, we examine the three DL classifiers identified in that review that are designed for 16S data. Fiannaca *et al*. (2018) propose a per-rank deep neural network with k-mer features, but the per-rank architecture introduces cascading error risks and limits scalability, and the model is trained on purely simulated reads. DERSI (Borgman *et al.* 2022) builds a CNN-based latent embedding space to denoise and classify sequences but does not explicitly model global dependencies or hierarchical taxonomy. Seq2Species (Busia *et al.* 2018) is a species-level classifier based on short-read deep neural networks, but it does not incorporate interpretability mechanisms or hierarchical inference and has not been benchmarked across diverse experimental datasets. The small number and limited scope of these tools show that, despite the importance of 16S sequencing in microbiome research, DL solutions tailored for 16S data remain underdeveloped. This deficiency further motivates DeepTaxa, which targets these limitations through a hybrid, interpretable architecture optimized for 16S data.

Building on the strengths and limitations of existing methods, we present DeepTaxa, a scalable framework for microbiome classification that combines CNNs for local-motif detection with a BERT-based transformer for global context modeling within 16S rRNA sequences. The combined architecture improves both accuracy and efficiency for microbiome taxonomic classification.

## Methods

DeepTaxa is a DL tool that performs multi-rank taxonomic classification of 16S rRNA sequences across the seven Linnean ranks within a single model. The architecture, HybridCNNBERTClassifier, captures local sequence motifs and long-range contextual dependencies in parallel. The model is trained with cross-entropy loss summed across the seven ranks with uniform per-rank weights; an alternative formulation using focal loss with rank-specific weighting was also evaluated, and cross-entropy yielded slightly higher species-level F1 across two independent seeds (see *Loss Function*). DeepTaxa is written in PyTorch, uses Hugging Face Transformers, and supports GPU mixed precision for training and inference. The command-line workflow takes FASTA sequences and corresponding taxonomic labels as input and does not depend on an external reference database at inference time (Fig. S1).

### Data preparation and tokenization

We used the Greengenes2 database (release gg_2024_09; [McDonald *et al.* 2024]), which contains 346 671 curated 16S rRNA gene sequences annotated across all main taxonomic ranks and comprising *N* sequences *S* = {*s*_1_, *s*_2_,…,*s_N_*} and corresponding taxonomic labels *Y* = {*y*_1_, *y*_2_,…,*y_N_*}, where each *y_i_* = [*y_i_*_0_, *y_i_*_1_,…,*y_i_*_6_] represents labels for the ranks R={domain, phylum, class, order, family, genus, species}. The dataset was partitioned into training (277 336 sequences, 80%) and test (69 335 sequences, 20%) sets using stratified sampling to preserve label distributions. The training set was further split into training (80%; 221 868 sequences) and validation (20%; 55 468 sequences) subsets. This single stratified split was used for all experiments; k-fold cross-validation was not performed due to the computational cost of training the hybrid architecture, though the large dataset size and stratified sampling provide reliable performance estimates. We curated a version of the Greengenes2 database in which short or undefined sequences were labeled as unclassified, enabling assessment of DeepTaxa performance on both defined and undefined taxonomic assignments. This curated dataset is publicly available from the Hugging Face Greengenes dataset repository under the terms of the Modified BSD License, as required by the original Greengenes2 license.

Each sequence was paired with a taxonomic label vector *y_i_* = [*y_i_*_0_, *y_i_*_1_, *y_i_*_2_,…,*y_i_*_6_], where *y_i_*_l_ represents the class index at the taxonomic level. Missing labels were mapped to a reserved integer to maintain rank order during training. Sequences were tokenized using the DNABERT-2 tokenizer (Zhou *et al.* 2023), which applies BPE to raw nucleotide sequences. Unlike fixed-length k-mer tokenizers used in earlier DNA language models, the DNABERT-2 BPE tokenizer uses a fixed 4096-token subword vocabulary, producing variable-length tokens that capture recurring DNA motifs. Each input 16S rRNA gene sequence is tokenized into BPE tokens and either truncated or padded to a fixed length of 512 tokens; for full-length sequences (approximately 1500 bp), this 512-token representation typically spans 800–1200 nucleotides depending on sequence composition. No sliding window or variable-region extraction is applied during preprocessing: the tokenizer processes each sequence from the 5’ end, and the 512-token context window captures the majority of the phylogenetically informative signal present in the full-length 16S gene. Labels are encoded as integers via rank-specific mappings, assigning “Unclassified” to missing entries to ensure complete dataset coverage.

### Model architectures

We developed a hybrid DL model for multi-rank taxonomic classification, designed to predict labels across all seven taxonomic ranks of 16S rRNA sequences (domain, phylum, class, order, family, genus, and species). The model combines two complementary feature extractors operating in parallel on the same tokenized input.

#### Multiscale hybrid CNN-BERT classifier

**Figure 1:**
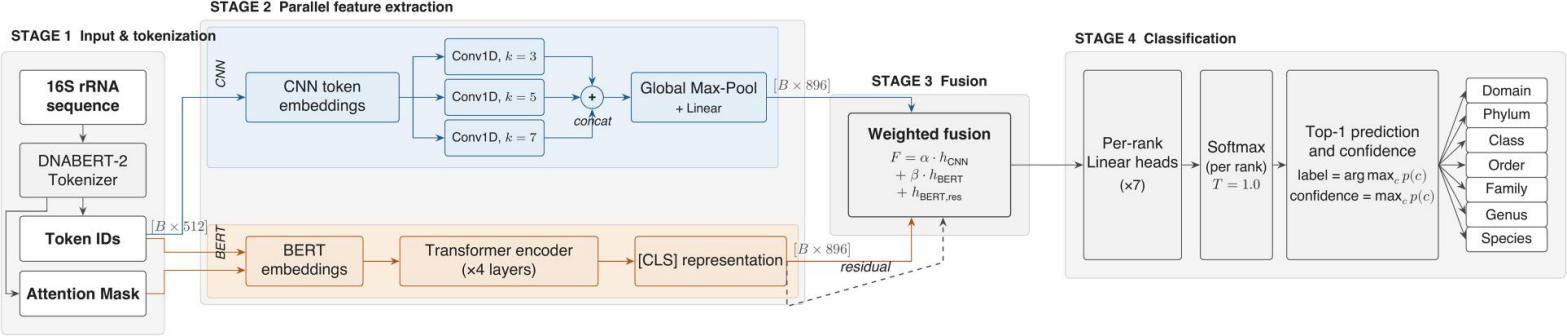
DeepTaxa hybrid CNN-BERT architecture for multi-rank taxonomic classification. Schematic of the HybridCNNBERTClassifier architecture, organized as four stages: input tokenization, parallel CNN and transformer feature extraction, feature fusion, and rank-specific classification. 16S rRNA sequences are tokenized using DNABERT-2 BPE tokenization and passed in parallel through a multiscale CNN branch and a compact transformer encoder trained from scratch. CNN and transformer representations are combined by learnable scalar weights with an additive BERT residual connection, then passed to seven parallel rank-specific classification heads for domain through species prediction. Layer dimensions, class counts, and hyperparameter values are reported in Methods and Table S1.

The HybridCNNBERTClassifier (Fig. 1) combines two pathways: a CNN (Kim 2014) for detecting short, conserved DNA motifs and a compact BERT-style transformer encoder for modeling long-range dependencies. Sequences are tokenized with the DNABERT-2 BPE tokenizer (Zhou *et al.* 2023) and then passed through both pathways in parallel. The CNN uses 896-dimensional token embeddings followed by multiscale convolutions with kernel sizes 3, 5, and 7 (256 filters per kernel), capturing local motifs across multiple receptive fields. The CNN features are projected to 896 dimensions to match the BERT hidden size and are combined with the BERT [CLS] representation using two learnable scalar weights that the optimizer adjusts during training. An additive BERT residual connection bypasses the weighted fusion. The transformer encoder (4 layers, 7 attention heads, 896 hidden size, 3584-dimensional feedforward intermediate) is trained from scratch on Greengenes2 16S rRNA sequences; DNABERT-2 contributes only the BPE vocabulary used to tokenize input sequences, not pretrained transformer weights. The fused representation is passed to seven parallel linear classifiers, one per taxonomic rank. This combination of local-motif extraction (CNN) and contextual modeling (transformer) yields gains over either component in isolation, as shown by the architectural ablation reported below.

#### Multi-rank classification strategy

Per-rank predictions are computed by applying softmax to the corresponding classifier head; the published checkpoints use uniform temperature *T* = 1.0 at every rank (no *post hoc* temperature scaling). This uniform-temperature choice gave better calibration than the per-rank temperature scaling used in an earlier development variant (phylum and class temperatures of 5.0; see *Calibration Analysis*). Prediction uncertainty at each rank is quantified using the entropy of the softmax distribution.

### Model training

The model was trained using the AdamW optimizer (Loshchilov and Hutter 2019) with hyperparameters set to $\beta_{1}=0.9$, $\beta_{2}=0.999$, $\varepsilon =1\times 10^{-8}$, and a weight decay of 0.01. We used a batch size of 64, a dropout rate of 0.2, and a warmup phase comprising 10% of the training steps. Training was conducted using mixed precision on an NVIDIA RTX 4090 GPU (24 GB VRAM) for 10 epochs. The training pipeline supports patience-based early stopping (configurable patience and minimum-delta thresholds); the published checkpoints use the final epoch-10 model because species-level validation F1 continued to improve through epoch 10 (Fig. S3), despite a mild increase in validation loss after epoch 7 (Fig. S2). Model parameter counts were 76.4 M for the full-length HybridCNNBERT checkpoint and 75.8 M for the dedicated V3–V4 amplicon checkpoint; the small difference is due to the smaller V3–V4 species label vocabulary (8347 versus 16 909 species in the full-length training set), with a partially offsetting reduction elsewhere in the V3–V4 model configuration. End-to-end training plus test-set prediction completed in approximately 1 h 20 min; the training portion averaged 7–8 min per epoch.

The full hyperparameter configuration is given in Table S1. We tuned these values with Optuna (Akiba *et al.* 2019), a Bayesian optimization framework, applying its Tree-structured Parzen Estimator (TPE) sampler to maximize validation accuracy across taxonomic ranks. Optuna pruned low-performance configurations (e.g., large kernel sizes with high computational cost); we further refined the learning rate through grid search to ensure stable behavior across initializations. The learning rate followed a linear warm-up over 10% of training steps and then decayed linearly to approximately 0 by the end of training. Throughout this manuscript, “compact” refers to the configuration in Table S1 (∼76 M parameters) adopted for all main results; “expanded” refers to an earlier ∼112 M-parameter Optuna variant retained only for the out-of-distribution comparative check reported in Results (*Similarity-Stratified Performance*).

### Loss function

DeepTaxa is trained with multi-rank cross-entropy loss using uniform per-rank weighting. For a single sequence, the loss at each taxonomic rank r is the standard cross-entropy


(1) $$L_{r}=-\text{log}\;p_{t,r}$$

where *p*${\;}_{t,r}$ is the predicted probability of the true class at rank *r*. The total loss aggregates the per-rank cross-entropy losses across all seven taxonomic ranks with uniform weights *w*${\;}_{r}=1$:


(2) $$L_{\text{total}}=\sum_{r=1}^{7}w_{r}L_{r}$$

We evaluated a focal-loss formulation with rank-specific weighting (γ=2.0 to 3.5; level weights [1.0, 1.5, 2.0, 2.5, 3.0, 4.0, 5.0] from domain to species) intended to emphasize harder, fine-grained classes. In a 2-by-2 factorial comparison (focal versus cross-entropy, with versus without rank weights) on two seeds (42 and 123), cross-entropy with uniform weights yielded a small but consistent species-level F1 advantage (mean +0.51 percentage points) and was adopted as the configuration.

### Evaluation metrics

Classification performance was assessed using accuracy, precision, recall, and F1-score at each taxonomic rank. All accuracy values reported in this study are top-1 accuracy, defined as the fraction of test sequences for which the single highest-probability predicted label exactly matches the ground truth taxonomy at the given rank; we do not report top-k accuracy for *k* > 1. Prediction uncertainty was evaluated using the entropy of the predicted distributions, and calibration quality was quantified using the expected calibration error (ECE) computed with 10 equal-width confidence bins.

### Evaluation of DeepTaxa on unseen taxonomic labels

To assess the DeepTaxa model’s generalization to unseen taxonomic labels, we evaluated its performance on the held-out test set. We classified sequences into two categories: “Seen” (species label present in the training vocabulary) and “Unseen” (species label absent from the training vocabulary). Evaluation was performed up to the rank preceding the first missing rank (e.g., up to family for a missing genus label). For each subset, we report the top-1 accuracy at each rank.

### Architectural ablation

To isolate the contribution of each architectural component, we trained four variants of DeepTaxa under the compact hyperparameter configuration (Table S1) on the same Greengenes2 2024.09 training set and evaluated each on the same held-out test set. The variants are: (i) the full HybridCNNBERT model with DNABERT-2 BPE tokenization and the BERT pathway active; (ii) a CNN-only variant with the BERT pathway removed and classification heads attached directly to the CNN output; (iii) a BERT-only variant with the CNN pathway removed and heads attached to the [CLS] representation of a randomly initialized 4-layer transformer; and (iv) a one-hot CNN variant in which DNABERT-2 BPE tokenization is replaced by raw one-hot nucleotide encodings, with the CNN pathway otherwise unchanged. All four variants share the same training schedule, optimizer, batch size, dropout, and seven parallel classification heads; only the indicated component is varied. Results are reported under the compact configuration with cross-entropy loss at seed 42 for a direct, reproducible comparison.

### Benchmarking against existing taxonomic classifiers

To evaluate DeepTaxa against established methods, we conducted a benchmarking study comparing its performance against widely used taxonomic classification tools, including DADA2 (Callahan *et al.* 2016), QIIME 2, Kraken2, and SINTAX, across all seven taxonomic ranks (from domain to species). DeepTaxa and the four established tools were evaluated on the same 69,335 full-length 16S rRNA test sequences from Greengenes2 (release 2024.09). The reference database and software versions used for each tool were as follows: QIIME 2 version 2024.5.0 with the q2-feature-classifier sklearn naive Bayesian plugin trained on the Greengenes2 2024.09 reference sequences; DADA2 version 1.30 (via Bioconductor in R 4.3.3) using the assignTaxonomy function with the Greengenes2 2024.09 training FASTA; Kraken2 version 2.1.3 with a custom database built from Greengenes2 2024.09 sequences via kraken2-build; and SINTAX via USEARCH v11.0.667 with the Greengenes2 2024.09 FASTA formatted according to SINTAX requirements. DeepTaxa predictions were generated using the published full-length checkpoint that was trained on the Greengenes2 2024.09 training set. All tools used the same underlying taxonomic reference to ensure a fair comparison. For each tool, we recorded per-rank top-1 accuracy on the 69,335-sequence test set. Wall-clock time for the full 69,335-sequence test-set classification was recorded for DeepTaxa (single GPU) and DADA2 (150 CPU cores) to enable the inference-cost comparison reported in Results.

## Results

We evaluated DeepTaxa by analyzing its training dynamics, per-rank classification metrics, sequence region importance, and embedding structure on both the validation and held-out test sets.

### Training and validation dynamics

We first examined the optimization trajectory of the HybridCNNBERT model during training. The model showed a substantial decrease in training loss across the 10 epochs, while the validation loss dropped from 3.0046 at epoch 1 to a minimum of 1.2552 at epoch 7, rising slightly to 1.3565 at epoch 10 (Fig. S2). The learning rate followed the linear warmup-and-decay schedule described in Methods, with a peak $5.0\times 10^{-4}$ at the end of warmup (10% of total steps) and decayed to approximately 0 by the end of training.

### Embedding structure of learned representations

**Figure 2:**
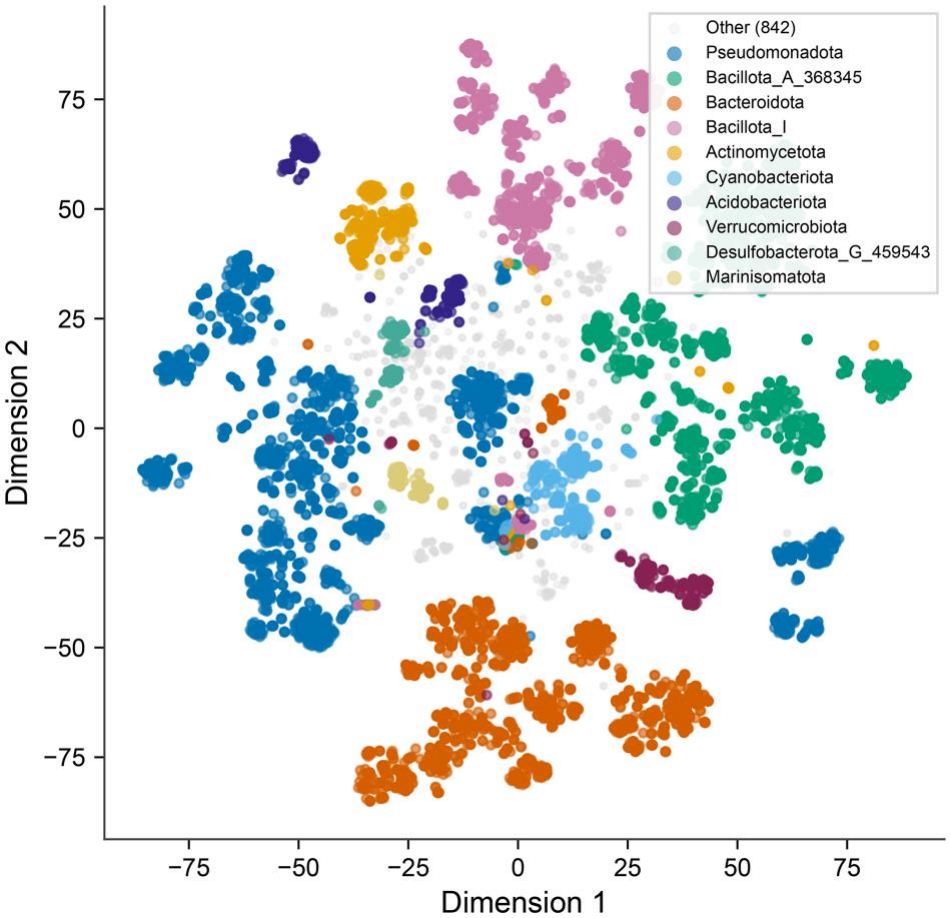
Phylum-level embedding structure of DeepTaxa sequence representations. Two-dimensional t-SNE projection of final-epoch embeddings for abundant phyla, showing partial taxonomic separation with overlap among some groups. A complementary species-level embedding is shown in Fig. S5.

To assess whether DeepTaxa learns taxonomically structured sequence representations, we visualized the final-epoch embeddings using t-SNE (Fig. 2). The phylum-level projection shows partial separation among abundant phyla, with some overlap among less-abundant groups. A complementary species-level visualization is shown in Fig. S5, where abundant species form more compact clusters. These visualizations support the interpretation that DeepTaxa learns taxonomically structured sequence representations, while also showing that two-dimensional projections are qualitative summaries rather than standalone evidence of classification performance.

### Performance across taxonomic ranks on validation set

Model performance improved across all taxonomic ranks throughout training (Fig. S4; the per-rank F1 trajectory is also shown as a heatmap in Fig. S3). High-level ranks (domain, phylum, and class) surpassed F1 scores of 0.99 on the validation set by epoch 2. Species-level performance, initially lower, rose markedly from 0.6667 at epoch 1 to 0.9198 at epoch 10. Genus and family followed similar trends on the validation set, reaching 0.9641 and 0.9846, respectively, by the final epoch.

### Performance across taxonomic ranks on test set

**Figure 3:**
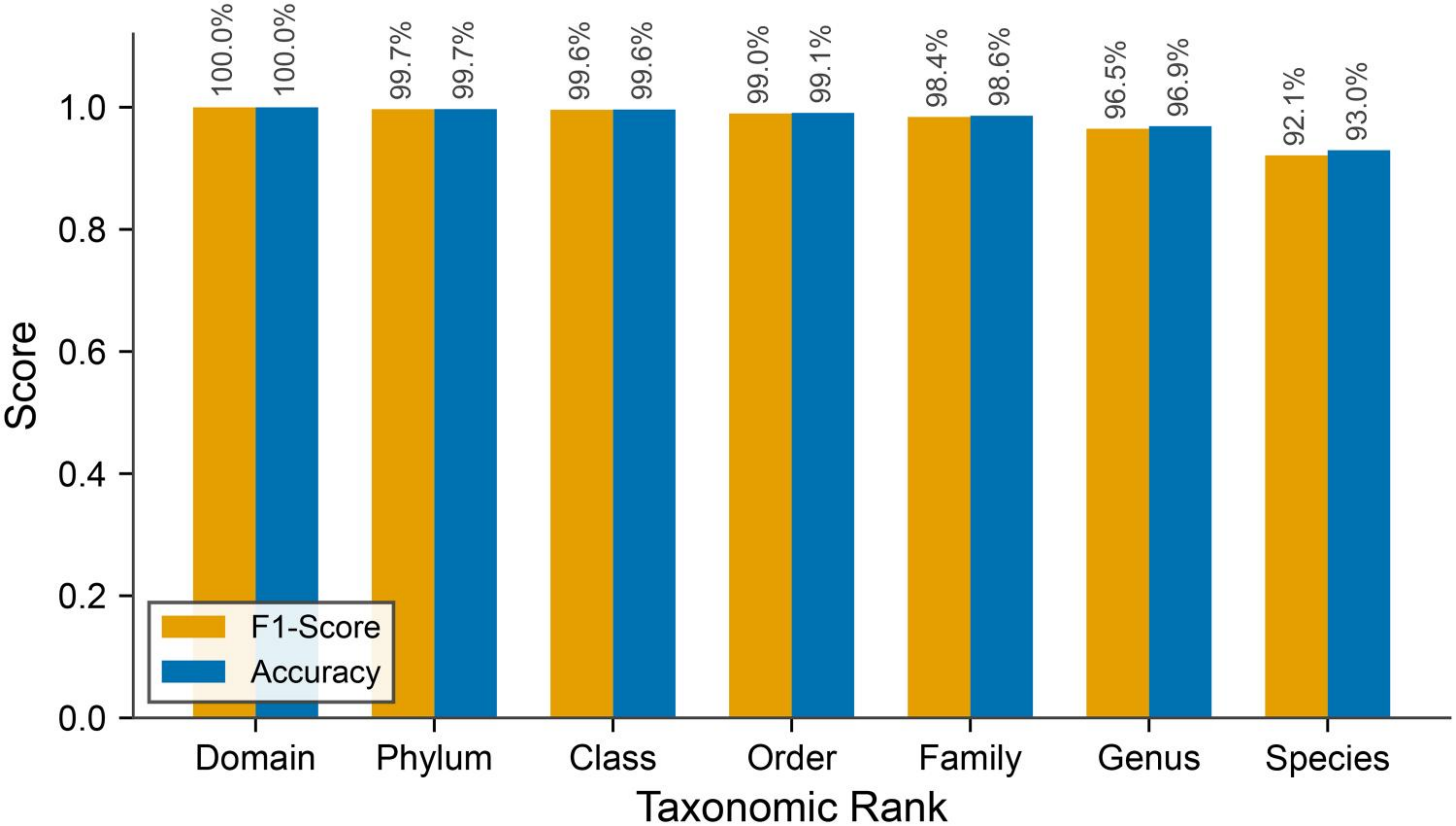
Per-rank test set performance. Grouped bar chart of weighted F1 score and accuracy at each of the seven taxonomic ranks on the held-out test set (69,335 sequences) for the full-length checkpoint. Bar heights show the 3-seed mean (seeds 42/123/456); cross-seed standard deviations are below 0.001 F1 at every rank (see the Multi-Seed Reproducibility subsection). DeepTaxa achieves species-level accuracy of 92.96% and species F1 of 0.9212, with accuracy and F1 above 0.99 at every rank from domain through class.

We evaluated the model on the held-out test set of 69 335 sequences. The numbers below are 3-seed means (seeds 42, 123, and 456). Domain achieved an F1-score and accuracy of 0.9998, while Species reached an F1 of 0.9212 and an accuracy of 0.9296 (Fig. 3). Intermediate ranks closely matched their validation performance: Phylum (F1 = 0.9968), Class (0.9959), and Genus (0.9648). Cross-seed standard deviations were at most 0.0008 F1 at any rank (species; ≤0.0003 F1 at every other rank), indicating high reproducibility; full per-rank means and standard deviations are reported in the *Multi-Seed Reproducibility* subsection below.

### Architectural ablation results

**Figure 4:**
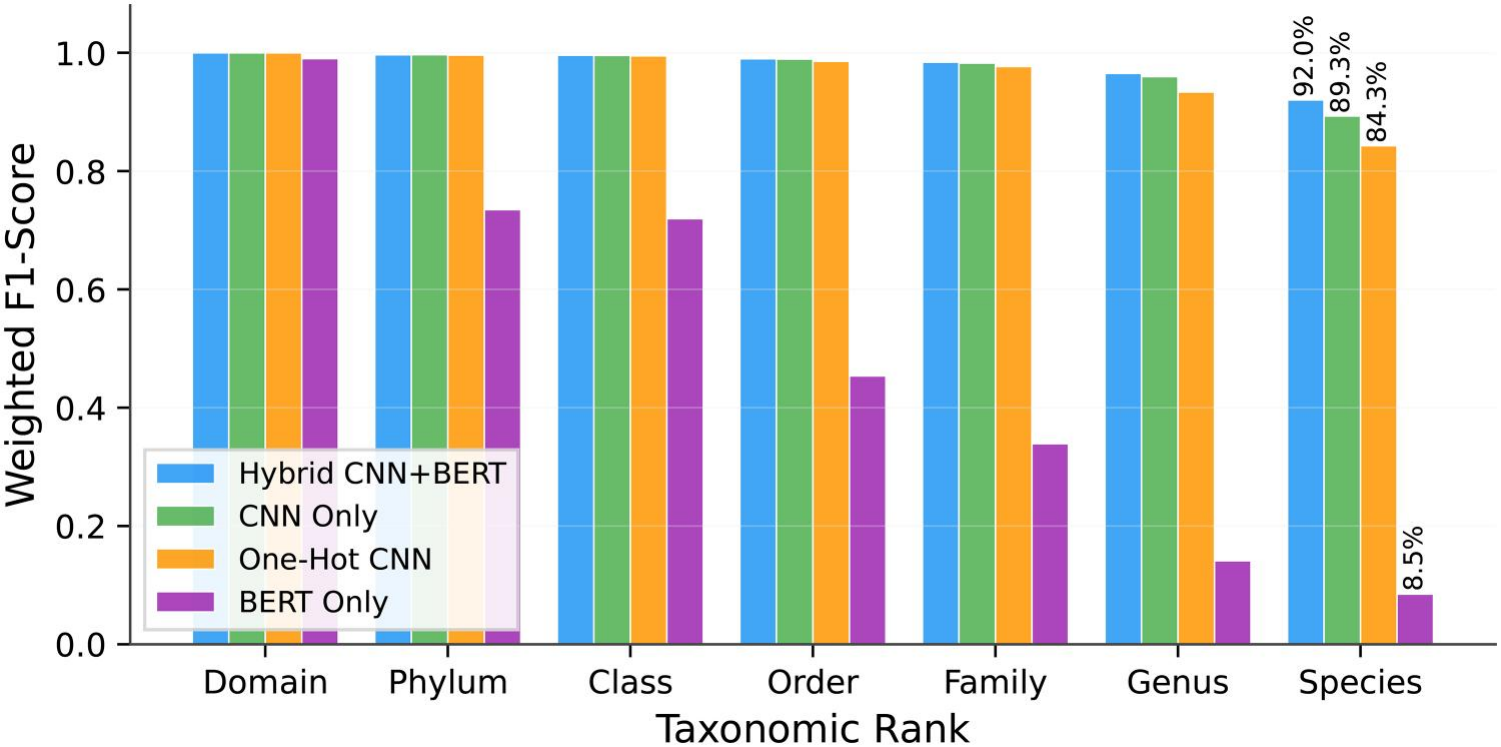
Architecture ablation of DeepTaxa. Per-rank weighted F1 comparison of four architecture variants trained under the compact configuration with cross-entropy loss and reported at seed 42: Hybrid CNN+BERT (species F1 0.9203), CNN-only (0.8931), One-hot CNN (0.8428), and BERT-only (0.0849). The hybrid gains 2.7 percentage points over CNN-only at species, and DNABERT-2 BPE contributes a further 5.0 percentage points over one-hot.

The architectural ablation isolates the contribution of each component under the compact configuration (Fig. 4). The full HybridCNNBERT model achieves a species F1 of 0.9203 (seed 42; the 3-seed mean of 0.9212 reported elsewhere covers seeds 42/123/456). Removing the BERT pathway (CNN-only) lowers species F1 to 0.8931, a 2.7 percentage-point drop attributable to the transformer’s contribution at fine-grained ranks. Replacing DNABERT-2 BPE tokenization with raw one-hot nucleotide encodings (one-hot CNN) lowers species F1 by a further 5.0 percentage points to 0.8428, indicating the value of subword-level input representations from the DNABERT-2 tokenizer. The BERT-only variant performs poorly (0.0849 species F1), indicating that a randomly initialized 4-layer transformer with the compact hyperparameters cannot learn the 16 909-class species head without the CNN’s multiscale local-pattern extraction. The CNN, therefore, carries the bulk of the discriminative signal; the transformer adds a measurable but smaller refinement; and the DNABERT-2 tokenization provides a substantial encoding-level gain over one-hot.

### Benchmarking against existing taxonomic classifiers

**Figure 5:**
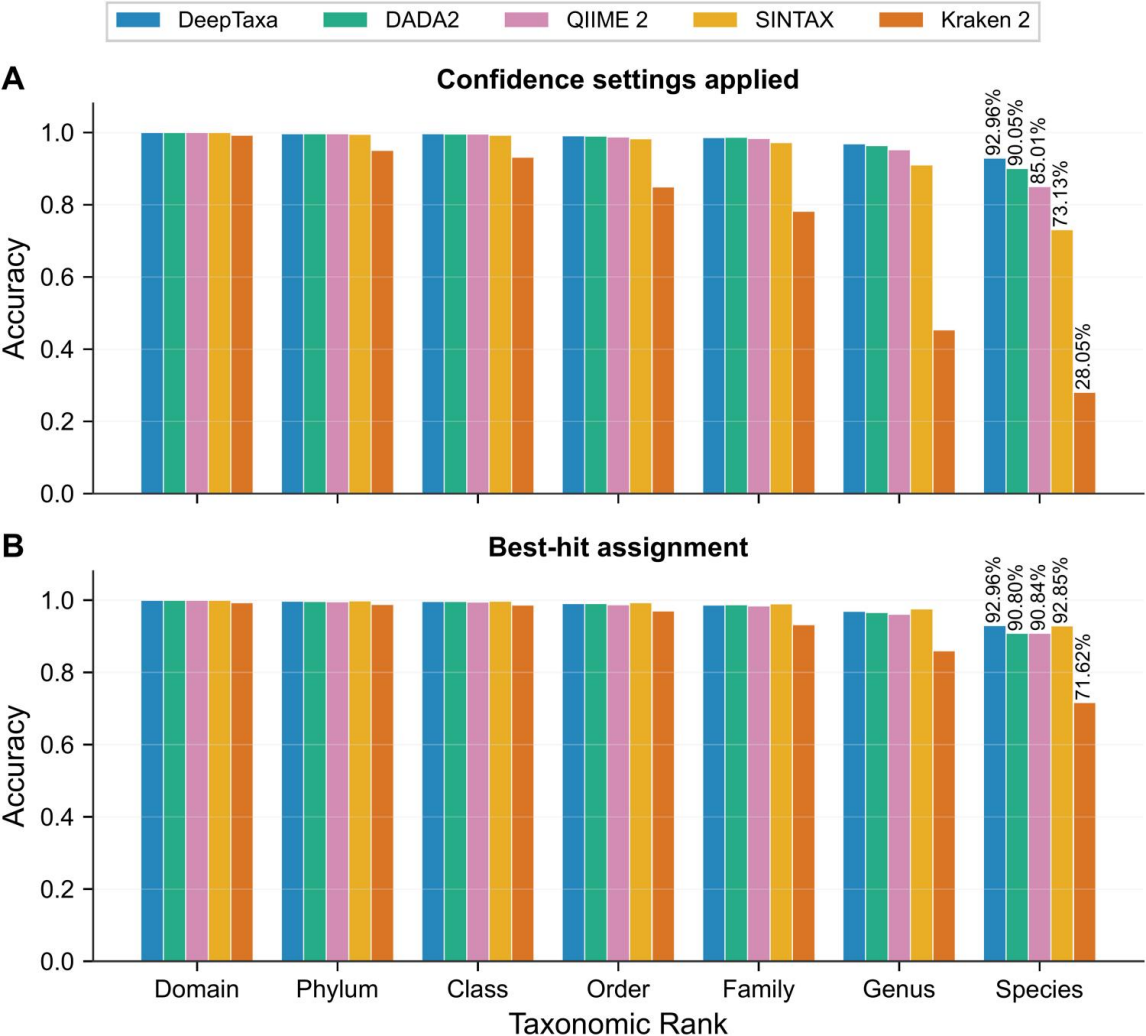
Benchmarking DeepTaxa against existing taxonomic classifiers. Per-rank classification accuracy for DeepTaxa and four established tools (DADA2, QIIME 2, SINTAX, Kraken 2) on the 69,335 full-length test sequences from Greengenes2 2024.09, under two scoring conventions. (A) Confidence settings applied: DADA2 and QIIME 2 at their defaults (minimum bootstrap 50; default classifier confidence), SINTAX at the recommended 0.8 bootstrap cutoff, and Kraken 2 at confidence 0.1. For DADA2, QIIME 2, and SINTAX, sub-threshold predictions are returned as unclassified and counted as incorrect; for Kraken 2 the confidence parameter sets the depth of the lowest-common-ancestor assignment (its software default is 0, and 0.1 is a common stricter setting). (B) Best-hit assignment: thresholding removed for all established tools (Kraken 2 at its software default of confidence 0), so each commits its single most likely call for every sequence, matching DeepTaxa’s argmax output. DeepTaxa returns a prediction for every sequence and is therefore identical in both panels. DeepTaxa leads at the species rank under both conventions, reaching 92.96% (3-seed mean) versus DADA2 90.05%, QIIME 2 85.01%, SINTAX 73.13%, and Kraken 2 28.05% in panel A, and remaining ahead under best-hit scoring in panel B (SINTAX 92.85%, QIIME 2 90.84%, DADA2 90.80%, Kraken 2 71.62%). DeepTaxa also classified the full test set in approximately 5 min on one GPU versus 3.9 h for DADA2 on 150 CPU cores.

To contextualize DeepTaxa’s performance, we benchmarked it against four widely used 16S rRNA taxonomic classification tools (DADA2, QIIME 2, Kraken 2, and SINTAX) across all seven taxonomic ranks under identical reference-database and test-set conditions (see *DeepTaxa Benchmarking against Existing Taxonomic Classifiers* in Methods). As shown in Fig. 5A, DeepTaxa is competitive across all ranks and leads at the species level, where most traditional tools underperform. On the held-out test set, DeepTaxa achieved a species-level classification accuracy of 92.96% and an F1-score of 0.9212 (3-seed mean), outperforming DADA2 (90.05%), QIIME 2 (85.01%), SINTAX (73.13%), and Kraken 2 (28.05%). At the genus level, DeepTaxa achieved 96.90% accuracy, ahead of DADA2 (96.36%), QIIME 2 (95.21%), SINTAX (91.04%), and Kraken 2 (45.35%). At the phylum level, DeepTaxa achieved 99.69%, comparable to DADA2 (99.65%), QIIME 2 (99.64%), SINTAX (99.51%), and Kraken 2 (95.04%). DADA2 is competitive at every rank above species and within ∼3 pp of DeepTaxa at species. SINTAX is competitive at the higher ranks; however, under its recommended 0.8 bootstrap-confidence cutoff, many species-level assignments fall below the threshold and are returned as unclassified, lowering its species accuracy to 73.13%. To confirm that these gaps reflect confidence filtering rather than an inability to reach the correct species, we also scored every tool by its single best hit with confidence filtering disabled (Figure 5B); under this matched convention, DeepTaxa remains first at the species rank (92.96), marginally ahead of SINTAX (92.85%) and above QIIME 2 (90.84%), DADA2 (90.80%), and Kraken 2 (71.62%). DeepTaxa’s balanced performance across all seven ranks, achieved while returning a prediction for every sequence, distinguishes it from reference-based classifiers such as DADA2 that lose ground at the species rank and from k-mer tools whose confidence settings leave many species-level calls unassigned. In addition to accuracy, DeepTaxa classified the full 69,335-sequence test set in approximately 5 min on a single GPU, compared to approximately 3.9 h for DADA2 on 150 CPU cores (Fig. 5), a lower wall-clock runtime under the tested hardware configuration at comparable or better species-level accuracy.

### Sequence region importance

To understand which portions of the 512-token input drive DeepTaxa’s predictions, we performed permutation importance analysis with 128-token windows. The 0–128 and 128–256 regions dominate the species- and genus-level signal (Fig. S9), with the 0–128 region contributing 0.333 to species-level accuracy when shuffled; the 256–384 and 384–512 regions contribute marginally, consistent with the 5’ localization of the V1-V2 hypervariable regions of 16S rRNA. Detailed numerical scores and an epoch-by-epoch view of how the regional importance evolves during training are reported in Fig. S8.

### Similarity-stratified performance

**Figure 6:**
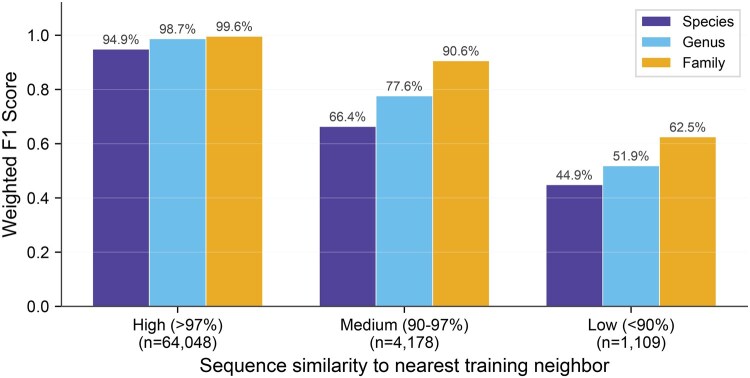
Similarity-stratified performance of DeepTaxa. Weighted F1 at species, genus, and family levels for test sequences bucketed by nearest-neighbor identity to the training set: high (>97%, *n* = 64 048; 92.4% of test set), medium (90%–97%, *n* = 4178), and low (<90%, *n* = 1109). High-bucket species F1 is 0.95, medium 0.66, low 0.45; family F1 stays ⩾ 0.90 through the medium bucket. A continuous view of accuracy as a function of nearest-neighbor identity, with Wilson 95% confidence intervals, is provided in Fig. S10.

To assess how performance varies with the similarity of test sequences to the training set, we used VSEARCH (—usearch_global) to compute the nearest-neighbor percent identity between each test sequence and the training pool, then partitioned the test set into three identity buckets and recomputed per-rank weighted F1 within each bucket using the same prediction pipeline used for the reported test-set metrics (Fig. 6; detailed values in Table S2; continuous identity trends in Fig. S10).

The vast majority of test sequences (92.4%) fall in the high-similarity bucket, where DeepTaxa attains species F1 of 0.95 and family F1 of 1.00. Performance declines smoothly with decreasing identity, but the species-level drop is steep: F1 falls from 0.95 in the high bucket to 0.45 in the low bucket. Family-level F1 stays above 0.90 through the medium bucket and falls to 0.63 in the low bucket. This pattern is the expected behavior of a supervised classifier operating on a finite training vocabulary: accurate species-level prediction requires that sufficiently similar sequences exist in the reference, while coarser ranks retain useful accuracy because their phylogenetic signal is broader. Figure S10 shows accuracy as a continuous function of nearest-neighbor identity with Wilson 95% confidence intervals, allowing readers to read off the expected accuracy for the identity distribution of their own data.

To verify that the compact configuration did not regress out-of-distribution performance relative to a larger Optuna-optimized variant, we recovered the prior expanded checkpoint (112.3 M parameters) from the public model repository and re-ran inference on the same test set through the same prediction path, then split the expanded predictions by the same identity buckets. The two architectures agree within 0.001 weighted F1 in every bucket (high: expanded 0.9492 versus compact 0.9488; medium: 0.6641 versus 0.6635; low: 0.4491 versus 0.4489) and within 0.004 accuracy. The compact configuration, therefore, matches the expanded variant at every identity stratum while requiring 32% fewer parameters and roughly half the training time.

### Performance on amplicon-length 16S sequences

To evaluate DeepTaxa on amplicon-length 16S data, we extracted *in silico* V3–V4 hypervariable-region amplicons from the Greengenes2 sequences using a degenerate-primer matching procedure (forward 341F = CCTACGGGNGGCWGCAG, reverse 805R = GACTACHVGGGTATCTAATCC; up to two mismatches per primer; median amplicon length 422 bp; extraction yields 98.4% on the training set and 98.5% on the test set). Applied zero-shot, the full-length checkpoint dropped from 92.96% to 60.7% species accuracy on the V3–V4 amplicons, consistent with the distribution shift from full-length to amplicon-length inputs. Training a dedicated V3–V4 model from scratch on the same training-set amplicons recovered species accuracy to 87.55% (seed 42; F1 = 0.8592, ECE = 0.0278), a gain of +26.9 percentage points over the zero-shot baseline. Per-rank performance of the seed-42 V3–V4 checkpoint is 99.99% (domain), 99.68% (phylum), 99.64% (class), 98.99% (order), 98.41% (family), 95.27% (genus), and 87.55% (species) accuracy. The V3–V4 model matches the full-length checkpoint to within 0.1 pp at the four highest ranks, falls slightly behind at family (−0.20 pp) and genus (−1.66 pp), and trails by 5.33 pp at species, consistent with the smaller information content of a ∼420 bp window relative to the full ∼1500 bp 16S gene.

A complementary fine-tuning experiment, in which the full-length checkpoint was used as initialization and training was continued on V3–V4 amplicons, underperformed training from scratch by approximately 3 pp at the species level. A V3–V4-specific Optuna search confirmed that the compact hyperparameters lie on a broad performance plateau in V3–V4 hyperparameter space (best Optuna run within +0.04 pp of the from-scratch configuration). The published V3–V4 checkpoint, therefore, uses the same compact HybridCNNBERT architecture as the full-length checkpoint, retrained from scratch on V3–V4 amplicons (see Methods, *Model Training*, for parameter counts and architectural details).

### Multi-seed reproducibility

To estimate the run-to-run variance of training, we trained the full-length checkpoint across three independent seeds (42, 123, 456) using identical hyperparameters. The per-rank mean accuracy/F1 values were 0.9998/0.9998 for domain, 0.9969/0.9968 for phylum, 0.9963/0.9959 for class, 0.9907/0.9897 for order, 0.9861/0.9841 for family, 0.9690/0.9648 for genus, and 0.9296/0.9212 for species. Across ranks, the cross-seed standard deviation was at most 0.0008 for F1 and 0.09 percentage points for accuracy. The training procedure is, therefore, highly reproducible at the seed level, and the published seed-42 checkpoint is statistically representative of the configuration. Because the V3–V4 checkpoint is the same architecture and training procedure applied to a different input distribution, we did not separately reproduce the multi-seed analysis at V3–V4 and report only the published seed-42 V3–V4 metrics elsewhere in this section.

## Discussion

Despite substantial progress in sequencing technologies and computational biology, taxonomic classification of microbial sequences from 16S rRNA data still struggles to achieve reliable species-level resolution and consistent assignments across phylogenetic ranks. The problem is compounded by the limited representation of microbial diversity in current reference databases, which constrains identification of novel or uncultured taxa. Most existing DL classifiers are optimized for whole-genome shotgun metagenomics (Liang *et al.* 2020; Mock *et al.* 2022), an approach that can be resource-intensive and is not always feasible. DeepTaxa addresses three gaps relevant to 16S applications: the effect of incomplete reference-database coverage, the need to predict multiple taxonomic ranks within a single model, and the absence of retrainable models suited to alternative training corpora. The hybrid CNN-BERT framework was designed for accuracy and retrainability on alternative training corpora.

### Performance accuracy and benchmarking

DeepTaxa achieved high classification accuracy across all seven taxonomic ranks on the Greengenes2 validation and test sets. The three highest ranks (domain, phylum, class) reached an F1 score above 0.99 by epoch 2 (Figs. S3 and S4). Genus and species, which carry larger label vocabularies and finer discriminative requirements, improved more gradually but reached a test-set F1 of 0.9648 (genus) and 0.9212 (species) for the full-length checkpoint (Fig. 3). The species rank, despite its 16,909-class output space, reached useful accuracy under the standard training schedule, and species F1 continued to rise across all 10 epochs (Fig. S3), indicating that further gains may be available with additional training.

DeepTaxa was benchmarked against four established tools: DADA2 (Callahan *et al.* 2016), QIIME 2 (Bolyen *et al.* 2019), Kraken 2 (Wood *et al.* 2019), and SINTAX (Edgar 2016). At the species level DeepTaxa leads (92.96% versus DADA2 90.05%, QIIME 2 85.01%, SINTAX 73.13%, and Kraken 2 28.05%; Fig. 5A), with DADA2 the strongest competitor, trailing by 2.91 percentage points. The gap narrows at coarser ranks, where the established tools approach DeepTaxa. . SINTAX’s lower species accuracy at its recommended 0.8 cutoff reflects confidence filtering rather than an intrinsic limitation of the method: when scored by best hit, it reaches 92.85% at species (Figure 5B), comparable to DeepTaxa. k-mer frequency captures less positional information than the convolutional and attention layers used here (Gao *et al.* 2017), but DeepTaxa’s main practical advantage is that it attains comparable accuracy while returning calibrated per-sequence confidence (species ECE 0.0242), so a high-confidence subset can be retained without the accuracy cost that thresholding imposes on the reference tools. DeepTaxa combines fine-grained discrimination at the species rank with high accuracy at every coarser rank.

### Multi-rank taxonomic classification

DeepTaxa’s multi-rank architecture jointly predicts all seven taxonomic levels, from domain to species, within a single model pass, using a shared sequence representation and seven parallel prediction heads. Although the heads operate independently without explicit hierarchical conditioning, joint optimization against a multi-rank cross-entropy loss with uniform per-rank weights pulls the shared representation toward features that are simultaneously useful for every rank, which in practice yields internally consistent lineage predictions and reduces obvious cross-rank contradictions. This design exploits the shared parent-rank signal in 16S sequences and is computationally efficient, since one forward pass produces all seven predictions.

### Generalization to unseen species labels

DeepTaxa retains useful parent-rank accuracy for sequences whose species labels were absent from training: for the 1,308 such test sequences, parent-rank accuracy declines monotonically from 99.8% at domain to 48.9% at genus (Fig. S7; full numbers in Results, *Performance on Unseen Taxonomic Labels*). The model relies on shared parent-rank signal rather than purely species-specific patterns, and the 48.9% genus accuracy on unseen species exceeds chance by a large margin even when the corresponding species is absent from training.

The complementary similarity-stratified analysis (Fig. 6 and Table S2) provides a continuous view of the same effect: weighted species F1 falls from 0.95 in the high-identity bucket (>97% identity to the nearest training sequence) to 0.66 at medium identity (90%–97%) and 0.45 at low identity (<90%), while family F1 stays above 0.90 through the medium bucket. Generalization is, therefore, not a binary property of “seen versus unseen species” but a continuous function of nearest-neighbor identity, and DeepTaxa’s coarser-rank predictions remain useful well into the regime where species-level prediction becomes unreliable.

### Architectural innovations and biological interpretability

At its core, DeepTaxa integrates a hybrid CNN-BERT architecture that combines multiscale convolutional filters with a compact transformer encoder trained from scratch using the DNABERT-2 BPE tokenizer (Zhou *et al.* 2023), which provides a fixed 4096-token nucleotide vocabulary built from multispecies sequences. The convolutional pathway captures local motifs across multiple receptive fields, while the transformer adds contextual refinement beyond what local features alone can provide. The architectural ablation reported in Results (*Architectural Ablation Results*; Fig. 4) supports a clear hierarchy of contributions under the compact configuration: the CNN pathway carries most of the discriminative signal, the transformer adds a measurable refinement at fine ranks, and a randomly initialized 4-layer transformer alone cannot learn the 16,909-class species head from BPE tokens without the CNN’s multiscale local-pattern extraction.

The choice of the DNABERT-2 BPE tokenizer over generic nucleotide tokenizations is deliberate. The DNABERT-2 tokenizer provides a 4096-token subword vocabulary built from multispecies nucleotide sequences and captures recurring k-mer-scale patterns that one-hot or fixed-k-mer encodings cannot represent compactly. General-purpose transformer-based 16S classifiers such as BERTax use the original BERT architecture pretrained on diverse metagenomic fragments spanning eukaryotes, viruses, bacteria, and archaea (Mock *et al.* 2022); this broad scope produces embeddings not explicitly optimized for the structural and functional characteristics of 16S rRNA sequences, namely their short length, conserved secondary structure, and taxonomically informative hypervariable regions. DeepMicrobes (Liang *et al.* 2020) is also trained on metagenomic data and does not explicitly model 16S-specific features. DeepTaxa instead trains its compact transformer encoder from scratch on prokaryotic 16S rRNA sequences using DNABERT-2 BPE tokenization; the architectural ablation (Fig. 4) confirms a 5.0 percentage-point species-level gain from DNABERT-2 BPE tokens over raw one-hot nucleotide encoding under matched CNN architecture, supporting the tokenizer choice.

We examined the model’s learned embeddings through clustering analysis. Two-dimensional t-SNE projections at epoch 10 show partial separation at the phylum level for the most abundant phyla, including *Pseudomonadota* and *Bacteroidota* (Fig. 2). A complementary species-level visualization shows more compact clustering for abundant species, including *Caldora sp010672925* and *Faecalibacterium duncaniae* (Fig. S5). The 2D silhouette score remained high at the species level (0.7377 at epoch 10) but near zero at the phylum level, indicating that the model’s species-level discriminative signal projects more cleanly into 2D than higher-rank distinctions. These findings indicate that DeepTaxa learns fine-grained taxonomic structure from sequences alone and suggest possible downstream applications in unsupervised sequence clustering and exploratory grouping of potentially novel or poorly represented taxa.

While clustering analysis demonstrates that DeepTaxa’s learned embeddings recover taxonomic structure, a finer-grained understanding of how specific sequence regions drive predictions is also needed. To interpret which sequence regions contribute most to DeepTaxa’s predictions, we conducted a permutation importance analysis with 128-token windows. It revealed that the model prioritizes the 0–128 and 128–256 sequence regions when making predictions at the species and genus levels (Fig. S9). These regions exhibited high importance scores, suggesting they harbor conserved or discriminative motifs relevant to fine-grained taxonomic classification, in line with the established role of the 5’ V1–V2 hypervariable regions of 16S rRNA. The deeper regions (256–384 and 384–512) contributed marginally, and domain-level scores remained uniformly low across all regions, consistent with domain prediction relying on broadly distributed signals rather than localized motifs. Future work can expand this analysis by identifying specific sequence motifs and evaluating their conservation across clades.

### Implementation and reproducibility

DeepTaxa is released as an open-source framework for 16S rRNA taxonomic classification and reproducible model development. The release includes source code, trained full-length and V3–V4 amplicon checkpoints, curated datasets, and workflows for training, prediction, and analysis. The full-length model has 76.4 million parameters and completed training and test-set prediction in approximately 1 h 20 min on a single NVIDIA RTX 4090 GPU. This resource profile makes the framework practical for laboratories with access to standard GPU computing resources, while retaining the flexibility to retrain the model on alternative taxonomic databases or amplicon regions.

### Limitations and future work

Several limitations should be noted. First, DeepTaxa was trained and evaluated primarily on Greengenes2; performance across other reference databases, such as SILVA and RDP, and across independent environmental or clinical datasets remains to be tested. Second, DeepTaxa currently operates on FASTA-formatted sequences and does not incorporate base-level quality scores, which may be relevant for noisy amplicon datasets. Third, the current study focuses on supervised multi-rank classification of prokaryotic 16S rRNA sequences, and additional work is needed to evaluate the framework on other marker genes or sequencing modalities, including 18S rRNA and shotgun metagenomic data. Although the embedding and permutation analyses provide qualitative insight into the learned representation, motif-level attribution and experimental validation of the most informative regions remain open directions for future work. Additional methodological limitations include the use of a single stratified train/validation/test split with three training seeds rather than k-fold cross-validation, the absence of retrained deep-learning classifiers with different target scopes from the benchmark, and timing comparisons limited to DeepTaxa and DADA2.

## Conclusion

DeepTaxa is a hybrid CNN-BERT deep learning framework for multi-rank taxonomic classification of 16S rRNA sequences. On the Greengenes2 2024.09 test set, the full-length checkpoint achieved a species-level F1 of 0.9212, a species-level expected calibration error of 0.0242, and F1 above 0.99 from domain through class, outperforming DADA2, QIIME 2, SINTAX, and Kraken 2 at the species rank while remaining competitive at higher ranks. The model retained useful parent-rank accuracy for sequences whose species labels were absent from the training set, and a similarity-stratified analysis showed that performance varied smoothly with decreasing identity to the training set rather than dropping at any single threshold. A dedicated V3–V4 amplicon checkpoint reached 87.55% species accuracy from an approximately 420 bp window, recovering most of the full-length performance. Source code, trained full-length and V3–V4 checkpoints, curated datasets, and reproducible workflows are publicly available.

## Supplementary Material

vbag166_Supplementary_Data

## Data Availability

The curated Greengenes2 dataset used for training, validation, and testing is available from the Hugging Face Greengenes dataset repository. The original Greengenes2 release (McDonald *et al.* 2024) is available under the terms of its Modified BSD License.

## Software availability

The DeepTaxa source code, trained checkpoints, curated datasets, reproducible workflows, and documentation are publicly available through the project GitHub, Hugging Face, and documentation repositories.
