# Supplementary material for: DeepTaxa: a hybrid CNN-BERT framework for 16S rRNA taxonomic classification
Source: Bioinform Adv. 2026 Jun 12;6(1):vbag166. doi: 10.1093/bioadv/vbag166 (PMC13316423; doi:10.1093/bioadv/vbag166)
Supplement: vbag166_Supplementary_Data [file vbag166_supplementary_data.zip › DeepTaxa_Supplementary_Material.pdf]

Supplementary Material for DeepTaxa: A Hybrid CNN-BERT Framework for 16S rRNA Taxonomic Classification

Rana Salah, Khlood R. AbdElaal, Lobna Ghonaim, Olaitan I. Awe, Ahmed Moustafa

The supplementary section below contains supplementary figures S1–S10 and extended methods and results that supplement the main text. All numerical results and conclusions reported in the main text are unchanged.

Supplementary Tables

Table S1: Hyperparameters used in DeepTaxa training. Values were determined through Optuna Bayesian optimization and grid search (see Methods).

Parameter	Value	Description
model_type	HybridCNNBERT	Hybrid CNN-BERT architecture
max_length	512	Max sequence length
batch_size	64	Samples per training batch
epochs	10	Training passes through dataset
learning_rate	0.0005	Initial learning rate
warmup_ratio	0.1	Warmup proportion of steps
embed_dim	896	Embedding dimensionality
num_filters	256	CNN filters per kernel
kernel_sizes	[3, 5, 7]	Multi-scale CNN kernels
num_conv_layers	1	CNN layer depth
dropout	0.2	Dropout probability
attention_heads	7	BERT attention heads
transformer_layers	4	BERT transformer layers
intermediate_size	3584	BERT FFN hidden size
loss_function	cross_entropy	Loss function (sum across ranks)
level_weights	[1.0, 1.0, 1.0, 1.0, 1.0, 1.0, 1.0]	Uniform per-rank loss weights
optimizer	AdamW	Optimizer with weight decay
weight_decay	0.01	L2 regularization
mixed_precision	Enabled	FP16 training acceleration

Table S2: Similarity-stratified test-set weighted F1 (compact, seed 42).

Bucket	Sequences	Family F1	Genus F1	Species F1
High (>97% identity)	64,048 (92.4%)	1.00	0.99	0.95
Medium (90–97%)	4,178 (6.0%)	0.91	0.78	0.66
Low (<90%)	1,109 (1.6%)	0.63	0.52	0.45

Table S3: Per-rank class statistics for the Greengenes2 2024.09 training set (277,336 sequences).

Rank	Unique taxa	Min support	Max support	Median support	Gini coefficient
Domain	2	1,798	275,538	n/a	0.49
Phylum	129	1	75,485	33	0.94
Class	349	1	58,177	13	0.96
Order	997	1	21,469	9	0.93
Family	2,250	1	19,368	7	0.91
Genus	7,287	1	5,966	5	0.84
Species	16,909	1	5,960	3	0.82

Supplementary Methods and Results

Sequence Region Importance Analysis

To identify which portions of the input sequence contribute most to taxonomic classification at each rank, we implemented a permutation importance analysis. The 512-token input sequence is divided into 4 non-overlapping contiguous regions of 128 tokens each (the default region width, `max_length / 4`; configurable via `--perm-region-size`). For each region, the BPE tokens within that region are randomly permuted (shuffled in place) while all other regions remain unchanged. The model's classification accuracy is then computed on the permuted input and compared to the accuracy on the original, unpermuted input. The importance score for region r at taxonomic rank k is defined as

$$\text{Importance}(r, k) = \text{Accuracy}_{\text{original}}(k) - \text{Accuracy}_{\text{permuted-r}}(k),$$

where the accuracy is computed over the full validation set. Higher scores indicate regions whose token content is more critical for classification at that rank. This procedure is repeated independently for each of the seven taxonomic ranks, yielding a 7-by-4 importance matrix per epoch at the default region width. The importance computation is performed at the end of each training epoch, enabling visualization of how region importance evolves during training (Supplementary Figure S8).

Performance on Unseen Taxonomic Labels

To further assess DeepTaxa's generalization beyond the closed-world setting, we evaluated its performance on unseen taxonomic labels, those present in the test set but absent from the training data. We split the test set into sequences whose species label was present in the training vocabulary (Seen, n = 68,027) and those whose species label was absent (Unseen, n = 1,308) and computed accuracy at every rank for each subset (Figure S7). For Unseen species (where species-level accuracy is 0% by construction), the model still classified higher ranks correctly: 99.8% at domain, 96.3% at phylum, 94.6% at class, 88.1% at order, 78.3% at family, and 48.9% at genus. For Seen species, accuracy at every rank was higher than for Unseen, ranging from 100.0% at domain to 94.8% at species, reflecting the additional difficulty of predicting unseen labels. The gradual decline of Unseen-species accuracy from domain through genus shows that DeepTaxa learns parent-rank and lineage-informative features rather than memorizing terminal labels: parent ranks of an unseen species can still be predicted reliably even when the species itself is absent from training.

Sequence Region Importance

To understand which regions of the 512-token input the model depends on, we performed permutation importance analysis with 128-token windows (four windows per sequence: 0–128, 128–256, 256–384, 384–512). At epoch 10, the 0–128 and 128–256 regions were most influential for fine-grained classification (Figure S9). At the species level, the importance score of the 0–128 region was 0.3330, followed by the 128–256 region at 0.1981. The final region (384–512) contributed minimally (<0.0002) across all ranks, and domain scores were consistently low (<0.006) across regions (Figure S9). This pattern recapitulates the known 5' localization of the V1–V2 hypervariable regions of 16S rRNA.

Focusing on the species level, the 0–128 region's importance increased over training while the deeper regions remained stable, indicating that the model progressively concentrates its discriminative signal in the most informative early portion of the sequence (Figure S8).

Clustering Analysis of Embeddings

To further assess the quality of the DeepTaxa embeddings, we performed a clustering analysis on the test set embeddings at Epochs 1 and 10, focusing on the top 10 most frequent phyla and species. We used t-SNE (van der Maaten and Hinton, 2008) for two-dimensional visualization and evaluated clustering performance using silhouette scores on the resulting 2D coordinates; silhouette scores measure how similar each point is to its own cluster compared to other clusters, with values ranging from -1 (poor clustering) to +1 (excellent clustering).

At the phylum level (Figure 2), the embeddings show partial separation, with distinct clusters for dominant phyla like *Pseudomonadota* (2,694 sequences) and *Bacteroidota* (1,780 sequences) but overlap among less-abundant phyla.

Species-level embeddings (Figure S5) reveal well-defined clusters, aligning with the model's focus on fine-grained features, with species such as *Caldora sp010672925* (203 sequences) forming tight clusters. Silhouette scores were computed on the 2D t-SNE projection restricted to the top-10 most abundant classes (for visual clarity, Figure S5 displays the top-15 species and Figure 2 displays the top-10 phyla): species-level cluster cohesion remained strong throughout training (0.7858 at epoch 1; 0.7377 at epoch 10), while phylum-level scores remained near zero (0.0385 at epoch 1; -0.0773 at epoch 10), reflecting that broad taxonomic groups do not project into compact 2D clusters even when the model classifies them with F1 above 0.99 (Figure 3). The discriminative signal at higher ranks is encoded along directions of the embedding space that 2D dimensionality reduction does not preserve.

Calibration Analysis

To assess whether DeepTaxa's softmax outputs provide a meaningful confidence signal, we measured the Expected Calibration Error (ECE) at every taxonomic rank using 10 equal-width confidence bins (see *Evaluation Metrics* in Methods). For the full-length checkpoint (3-seed mean), per-rank ECE is 0.0001 (domain), 0.0023 (phylum), 0.0024 (class), 0.0056 (order), 0.0075 (family), 0.0144 (genus), and 0.0242 (species). All ranks are well-calibrated under the 0.05 threshold commonly used in the literature, and standard deviations across the three seeds are below 0.001 ECE at every rank (Figure S6). Sequences classified with predicted probability above 0.95 achieve >0.98 accuracy at

the species level, while sequences below 0.80 confidence have substantially lower accuracy, confirming that the predicted probabilities can be used as usable confidence estimates. During development we identified and corrected a temperature-scaling artifact (per-rank temperatures of 5.0 at phylum and class) that had inflated the apparent ECE at those two ranks; the published checkpoints use uniform temperature T=1.0, after which the raw softmax outputs are well-calibrated at every rank without any post-hoc adjustment.

Class Imbalance and Per-Rank Taxon Counts

The Greengenes2 2024.09 training set is severely imbalanced at finer taxonomic ranks (Supplementary Table S3). The number of unique taxa rises from 2 at domain to 16,909 at species, and the per-class support distribution becomes increasingly long-tailed: the median number of training sequences per species is 3, while the most abundant species has 5,960. The Gini coefficient of the per-class support distribution rises from 0.49 at domain to 0.94 at phylum and remains at or above 0.82 at every rank below domain, confirming the long-tail structure.

The species rank is dominated by singletons: 44.8% of training species have exactly one training sequence. We additionally tested whether explicit class-weighted cross-entropy (inverse-frequency and square-root-inverse-frequency weighting) could improve the model's sensitivity to rare taxa, but neither weighting scheme produced a measurable species-F1 improvement over uniform weights. We attribute this to the BPE-token feature space provided by DNABERT-2 tokenization: subword units that recur across closely related taxa appear to provide enough cross-class regularization that explicit reweighting is unnecessary. The weighted-vs-macro F1 gap at species (0.9203 weighted vs 0.6755 macro, seed 42; 9,644 unique species classes appear in the test set) reflects the contribution of the long tail: singletons and small-support classes lower the macro mean substantially even when the model is well-calibrated on the bulk of the test distribution.

Together, these results establish DeepTaxa as a multi-rank taxonomic classifier for 16S rRNA data, with high accuracy across all seven ranks and benchmarking results that match or exceed four established tools at the species level. The next section discusses model behavior, limitations, and directions for further improvement.

Loss Function and Training Dynamics

We considered two loss formulations for the multi-rank classification objective: (i) cross-entropy with uniform per-rank weights, and (ii) focal loss with rank-specific weights designed to emphasize harder, fine-grained classes. Microbiome data are often skewed toward a few dominant taxa, making it difficult for models to learn from rare or underrepresented lineages; focal loss addresses class-imbalance settings of this kind by down-weighting easy, majority-class examples (Lin et al., 2020). To determine which formulation is better suited to multi-rank 16S classification, we ran a 2-by-2 factorial comparison (focal vs cross-entropy, with vs without rank weights) on two seeds. Cross-entropy with uniform weights yielded a small but consistent species-level F1 advantage (mean +0.51 percentage points), and we adopted it as the configuration. The result suggests that DNABERT-2-derived representations of 16S sequences are sufficiently informative that the gradient reweighting introduced by focal loss provides no additional benefit; the simpler cross-entropy formulation produces a slightly better optimum without requiring per-class or per-rank weighting heuristics.

Training and validation losses declined steadily over the 10 epochs; validation loss reached its minimum of 1.26 at epoch 7 and rose slightly to 1.36 by epoch 10 (Figure S2), indicating effective convergence with mild overfitting in the final epochs. The linear warmup-and-decay schedule (peak 5.0×10^{-4} at the end of warmup, decaying to approximately zero by the last training step), combined with careful tuning of optimization parameters, contributed to the early loss reduction. The species rank dominated the validation loss because of its 16,909-class output space, yet the multi-rank cross-entropy formulation with uniform per-rank weights produced consistent improvements at every rank without requiring rank-specific reweighting.

A concrete benefit of the cross-entropy switch beyond species F1 is calibration: the published checkpoint achieves species ECE of 0.0242 with no post-hoc temperature scaling and ECE below 0.0075 at every rank above family (Figure S6). An earlier prototype that applied per-rank temperature scaling with phylum and class temperatures of 5.0 had inflated those two ranks' ECE by two orders of magnitude despite leaving the underlying logits unchanged; the published configuration uses uniform T=1.0 and recovers near-perfect calibration at every rank. Calibration was also preserved when the input regime changed: the V3-V4 amplicon checkpoint (also trained with compact hyperparameters, from scratch on ~420 bp amplicons) achieves species ECE of 0.0278 (seed

42), within 0.004 of the full-length checkpoint despite the smaller input window and the smaller training-amplicon yield.

Supplementary Figures

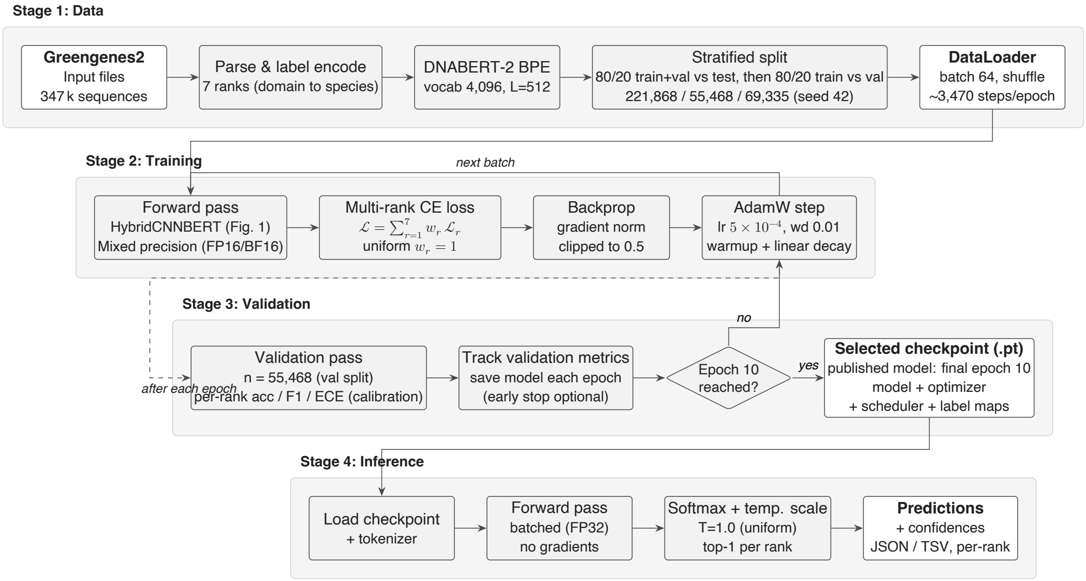

Figure S1: **DeepTaxa Training and Inference Workflow.** Greengenes2 16S rRNA sequences (~347 k) and taxonomy labels are parsed, label-encoded across seven taxonomic ranks, tokenized with the DNABERT-2 BPE tokenizer (vocabulary 4,096, maximum length L = 512), and partitioned by stratified sampling: first into training (80%) and test (69,335 sequences, 20%), then the training partition is further split into training (221,868) and validation (55,468) subsets (seed 42). The HybridCNNBERT model is trained with mixed precision (FP16/BF16), multi-rank cross-entropy loss (uniform per-rank weights $w_r = 1$), gradient-norm clipping at 0.5, and AdamW optimization (learning rate 5×10^{-4}, weight decay 0.01) with linear warmup followed by linear decay. After each epoch the model is evaluated on the validation split (per-rank accuracy, F1, and ECE); the pipeline supports patience-based early stopping (optional, disabled by default); the published checkpoints use the final epoch-10 model because species-level validation F1 continued to improve through epoch 10 (Figure S3), despite a mild increase in validation loss after epoch 7 (Figure S2). At inference time the checkpoint and tokenizer are loaded; sequences are processed by a batched no-gradient forward pass; per-rank softmax (temperature T = 1.0) followed by top-1 argmax produces the predicted label and softmax-derived confidence for each of the seven ranks; predictions and confidences are exported as JSON/TSV.

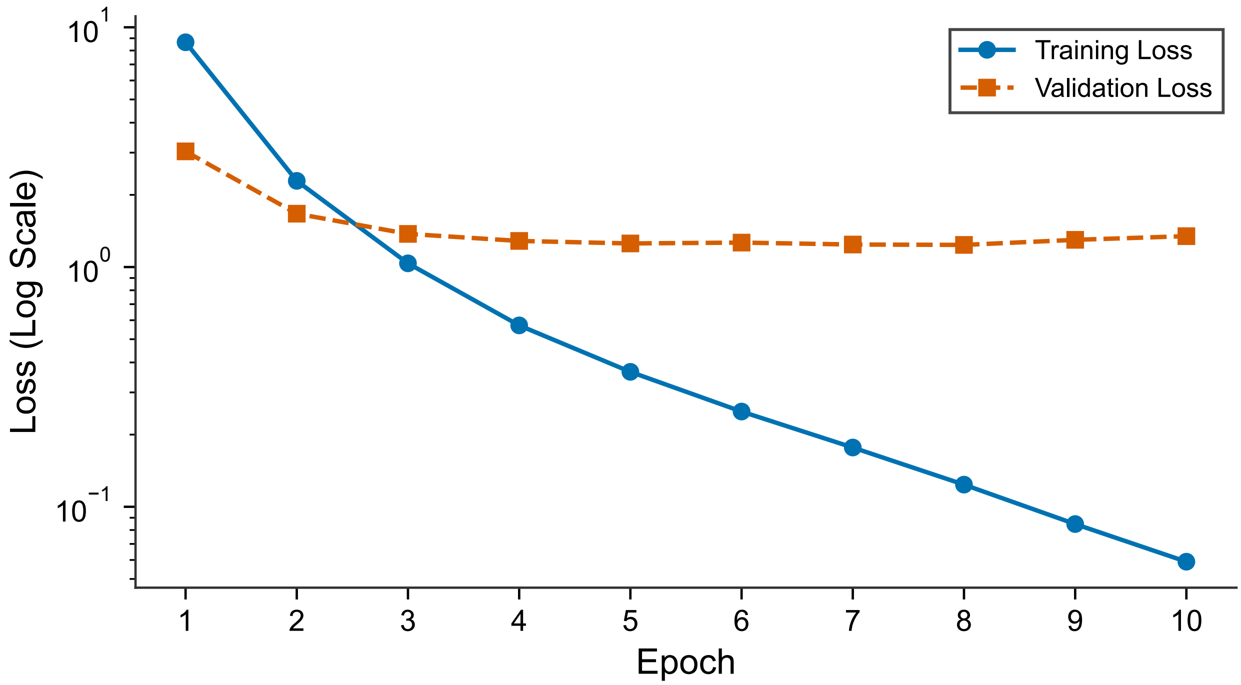

Figure S2: **Training and validation loss curves.** Training loss (solid) and validation loss (dashed) plotted on a logarithmic scale over 10 training epochs for the HybridCNNBERT full-length checkpoint on the Greengenes2 2024.09 dataset (277,336 training sequences). Training loss decreases monotonically; validation loss reaches its minimum (1.26) at epoch 7 and rises slightly to 1.36 by epoch 10, indicating effective convergence with mild overfitting in the final epochs.

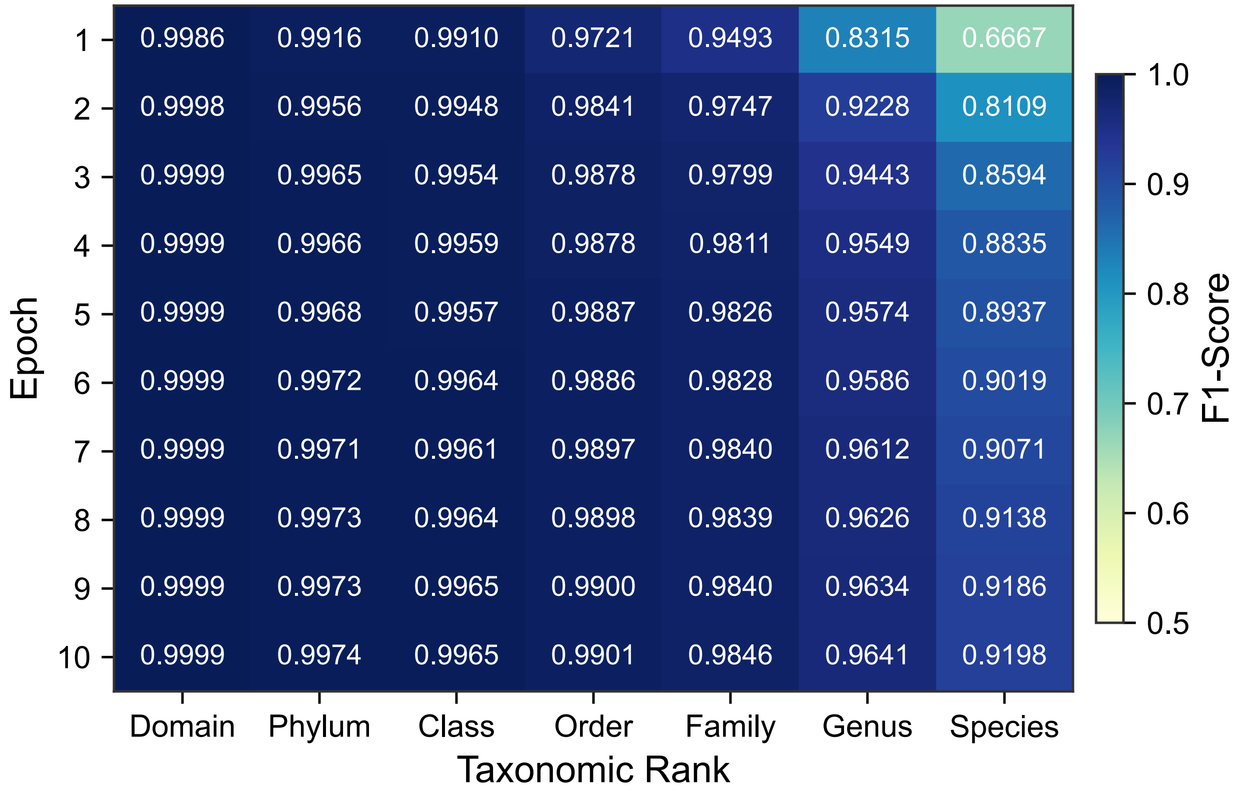

Figure S3: **F1 heatmap by epoch and taxonomic rank.** Per-rank weighted F1 on the validation set tracked across all 10 training epochs for the full-length checkpoint, illustrating the rank-by-rank convergence trajectory and the asymmetry between coarse-rank early saturation and species-rank gradual improvement.

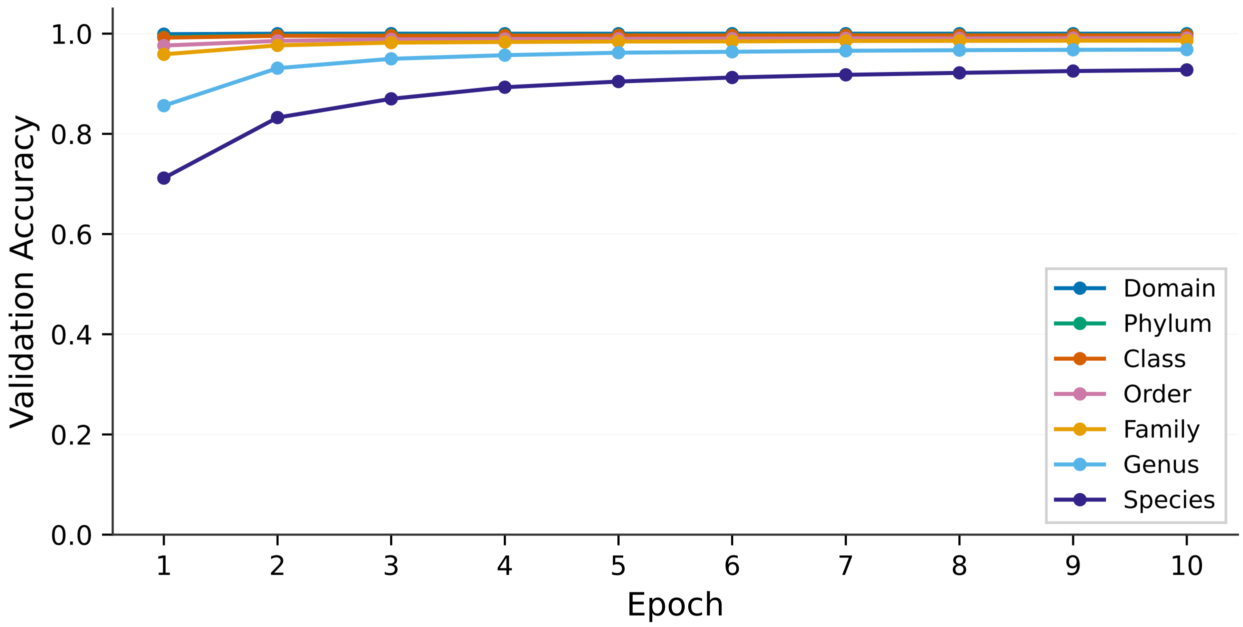

Figure S4: **Per-rank validation accuracy over epochs.** Validation-set top-1 accuracy at each of the seven taxonomic ranks across the 10 training epochs, showing monotone improvement at every rank with the largest gains at species and genus.

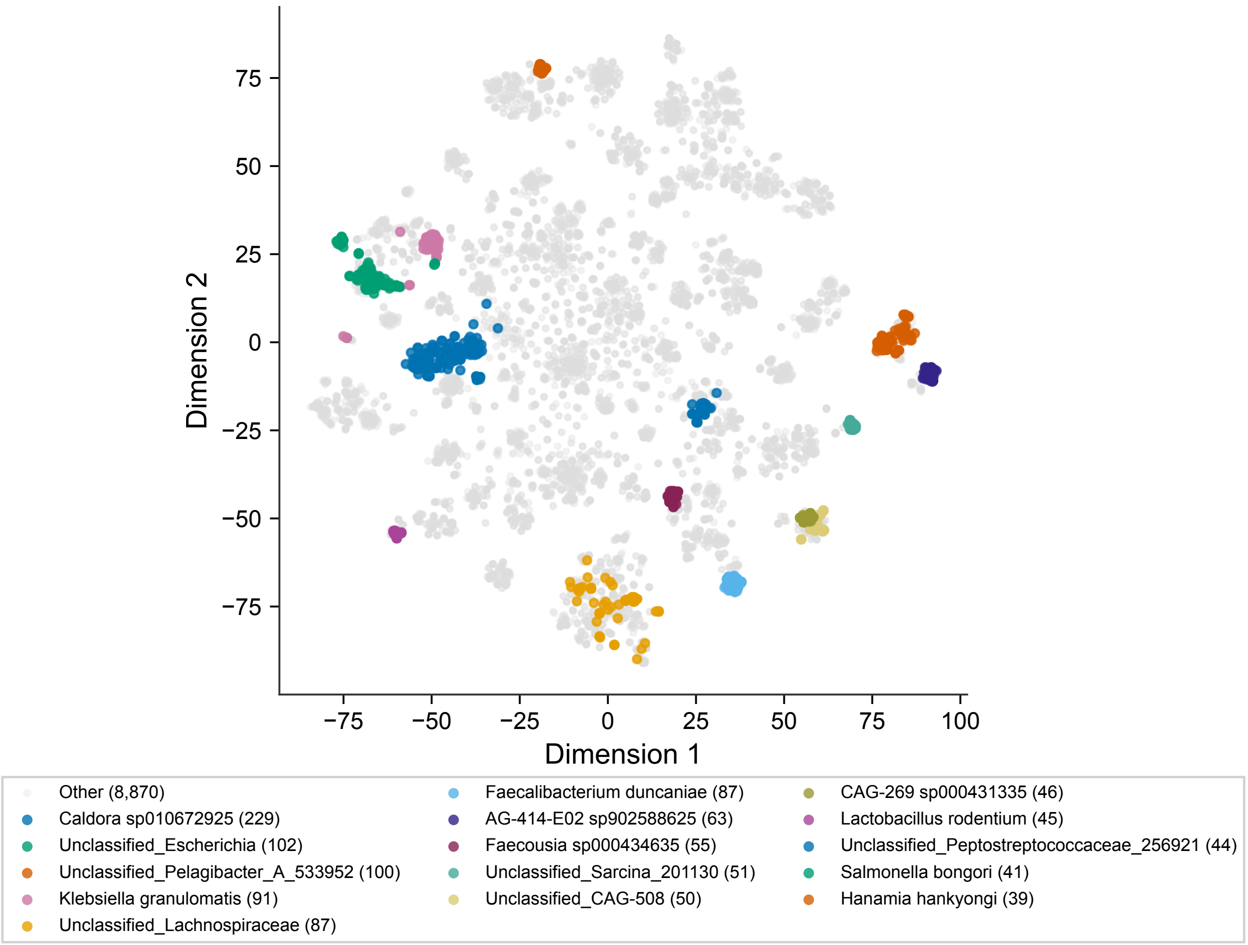

Figure S5: **t-SNE embedding visualization of the top-15 most frequent species at the final training epoch.** Two-dimensional t-SNE projection of the species-level embeddings, expanding the top-10 species view to include the next five abundant species. The silhouette scores reported in the Clustering Analysis of Embeddings subsection are computed on the top-10 subset of these classes for direct comparability with the phylum-level top-10 visualization in Figure 2.

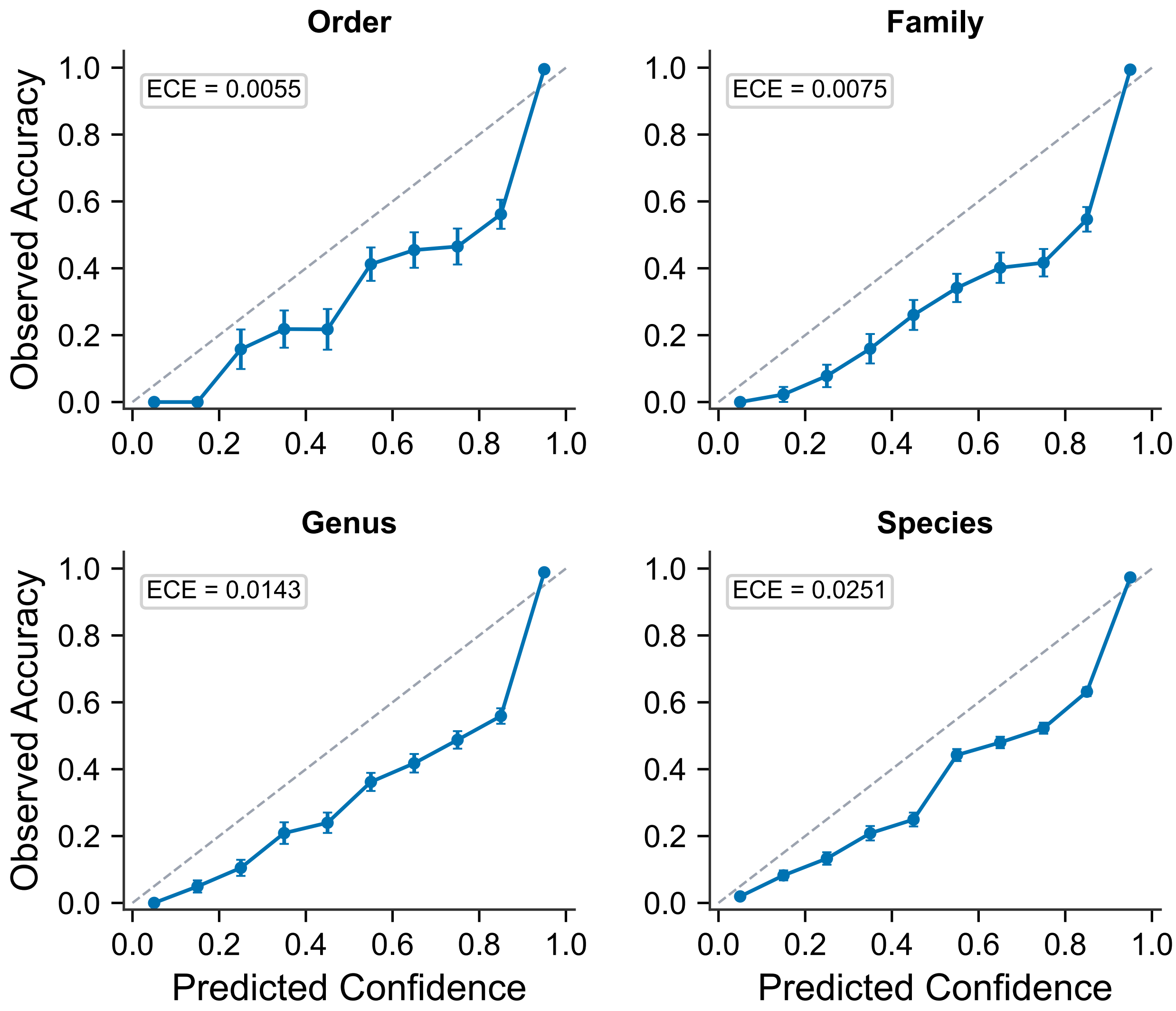

Figure S6: **Prediction calibration reliability diagrams.** Four-panel reliability diagrams (predicted confidence vs observed accuracy) for the seed-42 published full-length checkpoint at the four taxonomic ranks where calibration error is large enough to plot informatively (Order, Family, Genus, Species), with bin-count histograms shown beneath each panel. Domain, Phylum, and Class are omitted because their ECE values are below 0.003. The on-panel ECE values are seed-42 single-run values; the 3-seed-mean ECE values reported in the Calibration Analysis subsection differ from these by $\leq$0.001 ECE at every rank.

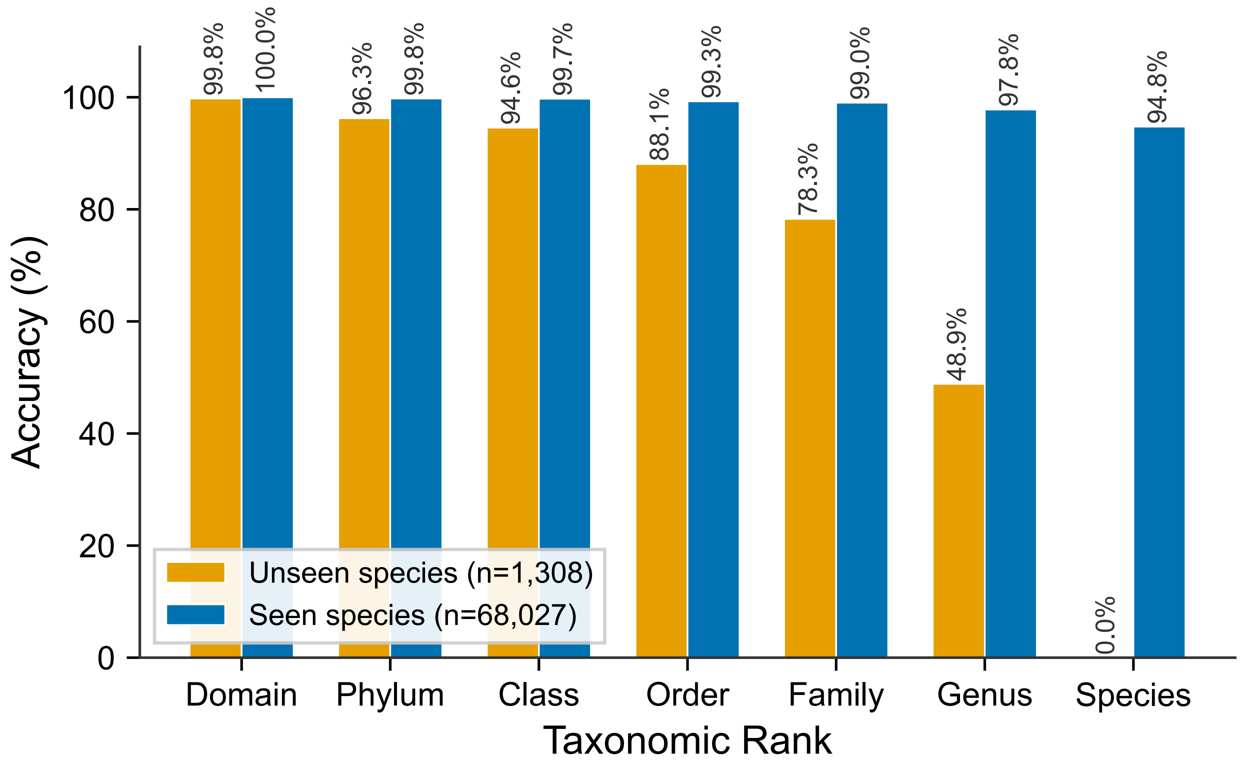

Figure S7: **Seen vs. unseen species accuracy.** Grouped bar chart comparing per-rank accuracy for test sequences whose species-level label was present (Seen, n = 68,027) or absent (Unseen, n = 1,308) in the training vocabulary. Unseen species achieve 0% species-level accuracy by definition, but the model correctly classifies them at higher ranks: 99.8% (domain), 96.3% (phylum), 94.6% (class), 88.1% (order), 78.3% (family), and 48.9% (genus). For seen species, accuracy at every rank is higher, ranging from 100.0% (domain) to 94.8% (species). The gradual decline of unseen-species accuracy from domain through genus indicates that DeepTaxa learns parent-rank and lineage-informative features rather than memorizing terminal species labels.

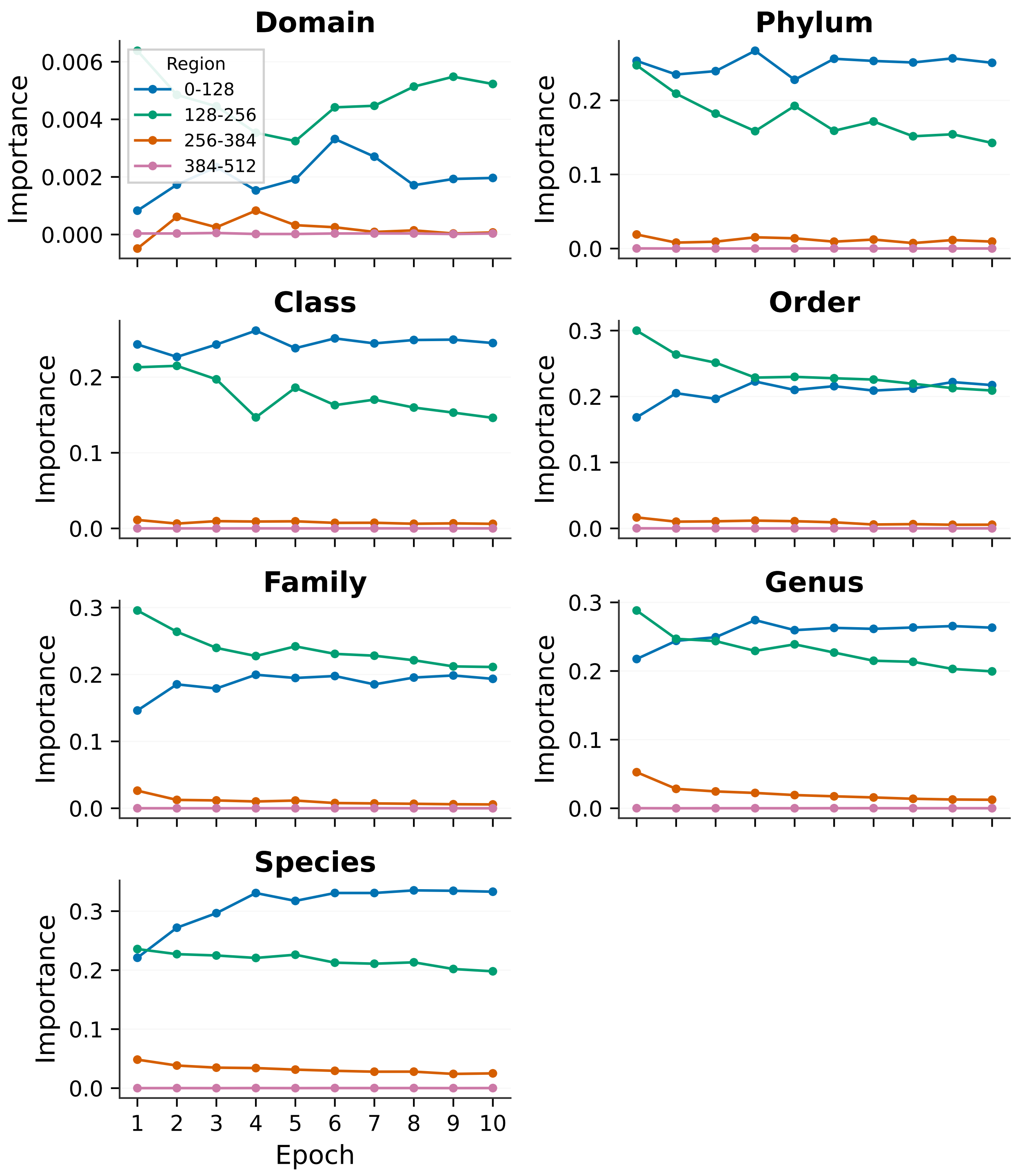

Figure S8: **Permutation importance trends for species classification across epochs.** Importance scores for the four 128-token sequence regions (0–128, 128–256, 256–384, 384–512 nt) tracked over 10 epochs. The 0–128 region rises across training, reaching 0.333 at epoch 10 (the value also reported as the 5' species cell in Figure S9). The 384–512 region remained near zero throughout training.

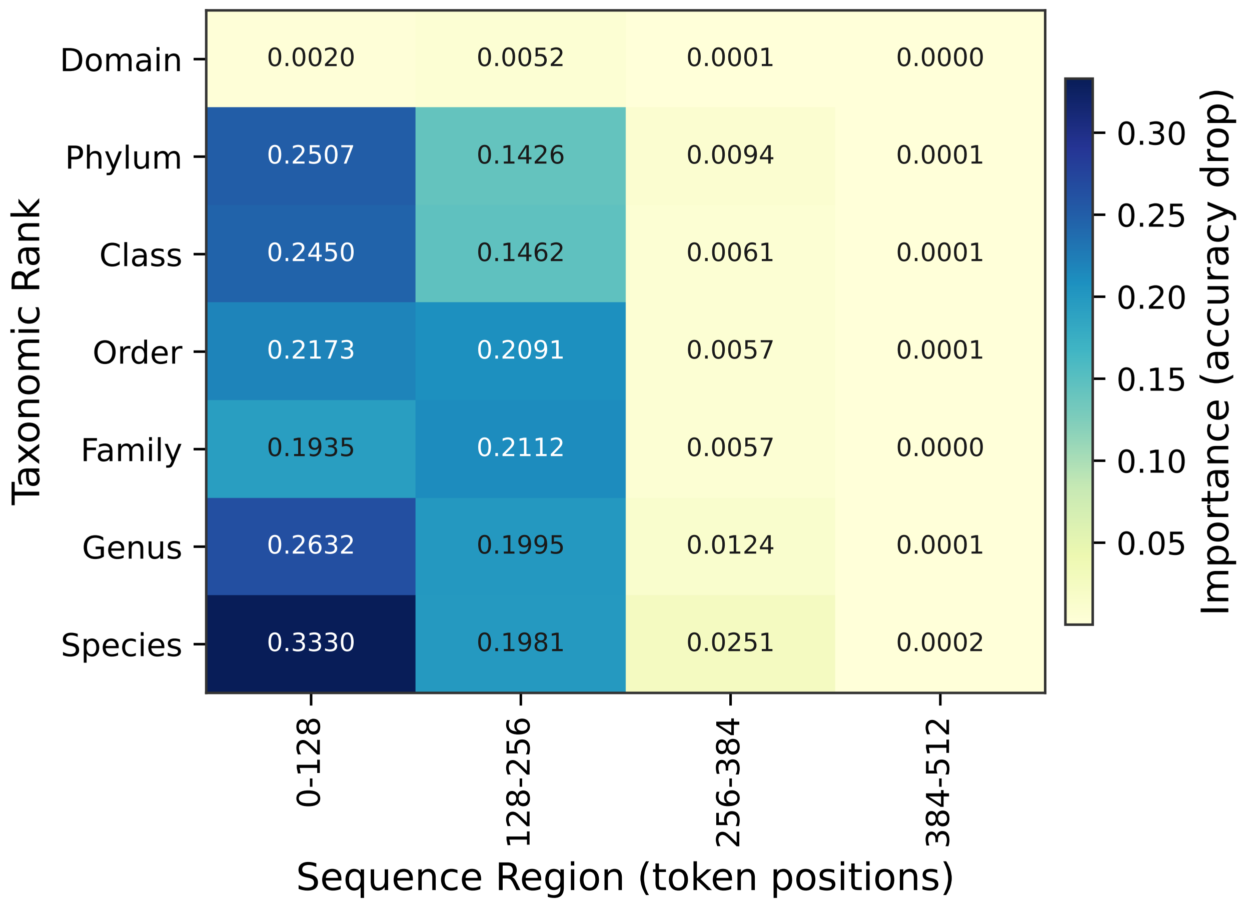

Figure S9: **Sequence region permutation importance.** Heatmap of permutation importance scores at epoch 10 across seven taxonomic ranks (rows) and four 128-token input regions (columns: positions 0–128, 128–256, 256–384, 384–512). Higher values indicate stronger accuracy drop when that region is permuted. The 0–128 region dominates species-level importance (0.333), followed by 128–256 (0.198); deeper regions and domain-rank scores are near zero.

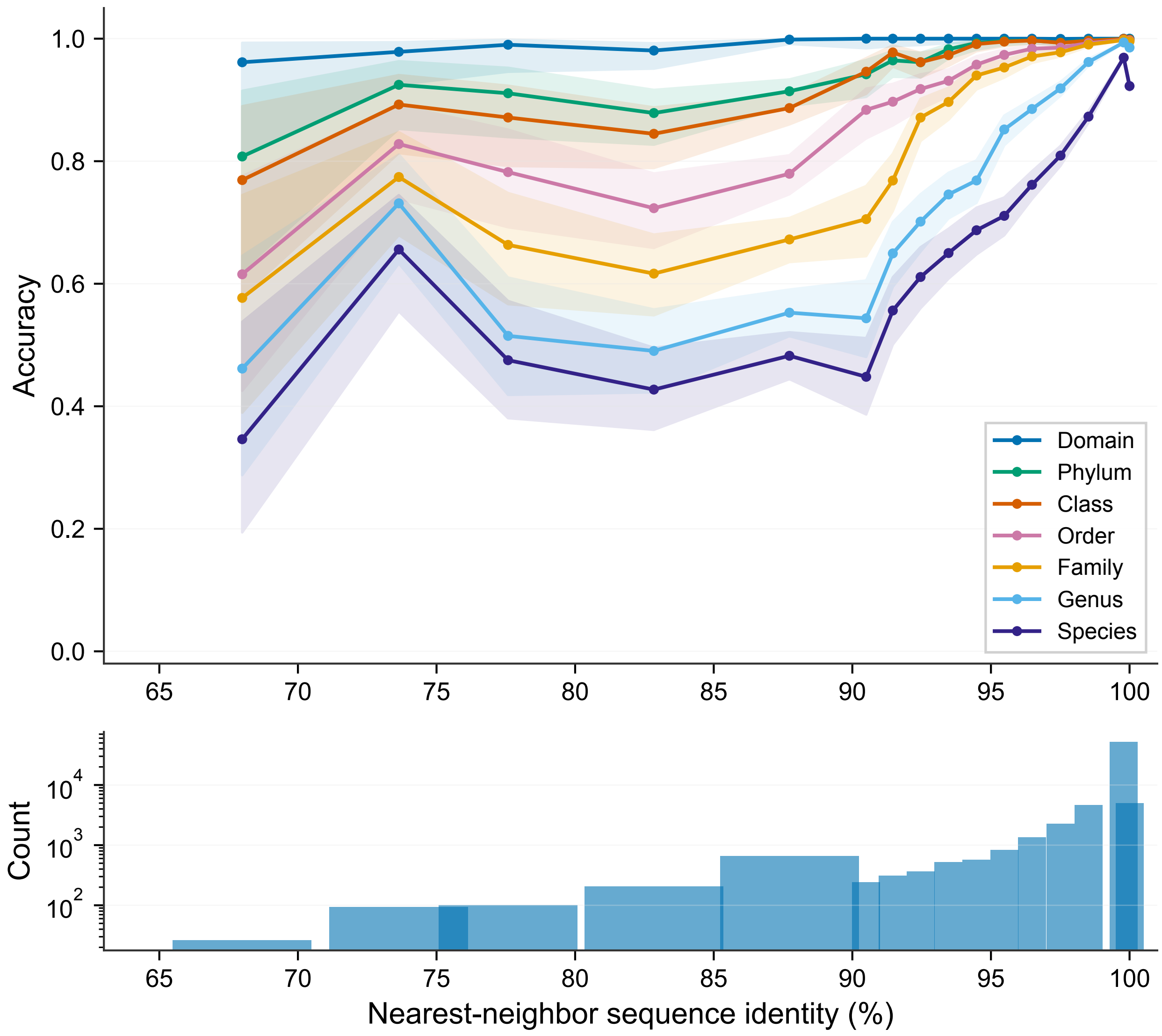

Figure S10: **Test-set accuracy as a continuous function of nearest-neighbor sequence identity.** Per-rank top-1 accuracy plotted against nearest-neighbor percent identity to the training set, with Wilson 95% confidence intervals computed in 1-percent-identity bins for the full-length checkpoint. The lower panel shows the sequence count in each bin on a logarithmic scale. The curves complement the discrete-bucket view of Figure 6 by showing the smooth decline of species-level accuracy as identity falls below approximately 97%, while coarser ranks remain more stable.

References

T.-Y. Lin, P. Goyal, R. Girshick, K. He, and P. Dollár. Focal loss for dense object detection. *IEEE Transactions on Pattern Analysis and Machine Intelligence*, 42(2):318–327, 2020. doi: 10.1109/TPAMI.2018.2858826. PMID: 30040631.

L. van der Maaten and G. Hinton. Visualizing data using t-sne. *Journal of Machine Learning Research*, 9:2579–2605, 2008. URL `https://jmlr.org/papers/v9/vandermaaten08a.html`.
